# Supplementary material for: Implementation of a multicomponent family support intervention in adult intensive care units: study protocol for an embedded mixed-methods multiple case study (FICUS implementation study)
Source: BMJ Open. 2023 Aug 8;13(8):e074142. doi: 10.1136/bmjopen-2023-074142 (PMC10414125; doi:10.1136/bmjopen-2023-074142)
Supplement: Supplementary data [file bmjopen-2023-074142supp001.pdf]

Summary of the study endpoints, definition, data source operationalization and data collection timepoints

STUDY ENDPOINTS	DEFINITION	DATA SOURCE	OPERATIONALIZATION	DATA COLLECTION TIMEPOINT T1	T2	T3	T4
**Implementation Process**							
Process indicators	Course of integration of FSI in team / care delivery processes and observed integration mechanisms	KP	Small group interviews[1]	x		x	
		KP, HP	NoMAD [1, 2, 3]			x	x
		KP	Implementation rating tool [1]	x		x	
**Implementation Outcomes**							
**Fidelity: Delivery**							
Relative frequency	Number of interventions per length of stay (LoS)	-	Intervention fidelity log[1,9]	x	x	x	x
Absolute dose	Total minutes of interventions	-	Intervention fidelity log[1,9]	x	x	x	x
Consistency	Consistent delivery of the FSI within and across ICUs as defined per intervention manual	-	Intervention fidelity log[1,9]	x	x	x	x
Availability	Availability of FN (days) as defined per trial protocol to ensure intervention capacity	KP	Intervention capacity assessment[1]	x			
		FN	Focus group interviews[2]				x
**Fidelity: Receipt**							
Attendance at case conferences	Overall participation rate	FN, (LI)	Case conferences[1,4]	x	x	x	x
Comprehension & skills	Skills in working systematically with families	FN	FNPS[1, 5]	x	x		x
		FN	Focus group interviews[2]				x
**Fidelity Enactment**							
Engagement & performance	Quality and consistency of intervention performance	FN	Fidelity Self-Rating Tool[1]		x		x
		Researcher, FN	Semi-structured observation[1]		x		
		FN	Focus group interviews[2]				x
**Feasibility**	Extent to which the FSI can be successfully used and carried out within ICU settings	KP	FIM[1,2,6]	x		x	x
		KP, HP	Focus group interviews[2]				x
**Acceptability**	Perception among clinical partners that the FSI is agreeable	KP	AIM[1,2,7]	x		x	x
		KP, HP	Focus group interviews				x
**Appropriateness**	Perceived fit, relevance and compatibility of the FSI for clinical partners	KP, HP	IAM[1,2,8]	x		x	x
		KP, HP	Focus group interviews[2]				x
**Sustainability / Sustainment**	Existence of FN role assessed 6 and 12 months after study end How likely the FSI is to be sustained in the future with necessary structures and processes in place to maintain FSI in ICUs	KP	Sustainability implementation survey[10]				x
		KP; HP	Focus group interviews[2]				x
**Contextual Determinants**	Influencing barriers and facilitators as conceptualized in CFIR and NPT	KP	Small group interviews[1]	x		x	
		KP, HP	Focus group interviews[2]				x

Abbreviations: FN Family Nurses, LI local implementers, KP Key clinical partners, HP Health Care Professionals, ICU Intensive Care Unit, LoS Length of stay, CFIR Consolidated Framework for Implementation Research, NPT Normalization Process Theory

[1] Part of the process evaluation
[2] Part of the summative evaluation
[3] German Version of the Normalization Measure Development Questionnaire (NoMAD) (64)
[4] Monthly case conferences throughout the 18-months active implementation and delivery phase, attendance is mandatory for FN, voluntary for LI
[5] German version of the Family Nursing Practice Scale (FNPS) (65)
[6] German version of the Feasibility Intervention Measure (FIM) (66)
[7] German version of the Acceptability Intervention Measure (AIM) (66)
[8] German version of the Intervention Appropriateness Measure (66)
[9] Case report forms embedded in RED Cap (67) and completed throughout the study by family nurses / interventionists
[10] Data collection occurs 6 and 12 months after active implementation and intervention delivery / FICUS study end
