# Supplementary material for: Implementation of a multicomponent family support intervention in adult intensive care units: study protocol for an embedded mixed-methods multiple case study (FICUS implementation study)
Source: BMJ Open. 2023 Aug 8;13(8):e074142. doi: 10.1136/bmjopen-2023-074142 (PMC10414125; doi:10.1136/bmjopen-2023-074142)
Supplement: Author's manuscript [file bmjopen-2023-074142.draft_revisions.pdf]

BMJ Open

BMJ Open is committed to open peer review. As part of this commitment we make the peer review history of every article we publish publicly available.

When an article is published we post the peer reviewers' comments and the authors' responses online. We also post the versions of the paper that were used during peer review. These are the versions that the peer review comments apply to.

The versions of the paper that follow are the versions that were submitted during the peer review process. They are not the versions of record or the final published versions. They should not be cited or distributed as the published version of this manuscript.

BMJ Open is an open access journal and the full, final, typeset and author-corrected version of record of the manuscript is available on our site with no access controls, subscription charges or pay-per-view fees (http://bmjopen.bmj.com).

If you have any questions on BMJ Open's open peer review process please email info.bmjopen@bmj.com

BMJ Open

Implementation of a multicomponent family support intervention in adult intensive care units – Study protocol for an embedded mixed methods multiple case study (FICUS Implementation Study)

Journal:	*BMJ Open*
Manuscript ID	bmjopen-2023-074142
Article Type:	Protocol
Date Submitted by the Author:	28-Mar-2023

Complete List of Authors:	Oesch, Saskia; University of Zurich Faculty of Medicine, Institute for Implementation Science in Health Care; University Hospital Zurich, Center of Clinical Nursing Science<br>Verweij, Lotte; University of Zurich Faculty of Medicine, Institute for Implementation Science in Health Care; University Hospital Zurich, Center of Clinical Nursing Science<br>Clack, Lauren; University of Zurich Faculty of Medicine, Institute for Implementation Science in Health Care; University Hospital Zurich, Division of Infectious Diseases and Hospital Epidemiology<br>Finch, Tracy; Northumbria University, Nursing, Midwifery and Health<br>Riguzzi, Marco; University of Zurich Faculty of Medicine, Institute for Implementation Science in Health Care; University Hospital Zurich, Center of Clinical Nursing Science<br>Naef, Rahel; University of Zurich Faculty of Medicine, Institute for Implementation Science in Health Care; University Hospital Zurich, Center of Clinical Nursing Science

Keywords:	Adult intensive & critical care < INTENSIVE & CRITICAL CARE, Nursing Care, Protocols & guidelines < HEALTH SERVICES ADMINISTRATION & MANAGEMENT

SCHOLARONE™ Manuscripts

Implementation of a multicomponent family support intervention in adult intensive care units – Study protocol for an embedded mixed methods multiple case study (FICUS Implementation Study)

Saskia Oesch RN MSc[1, 2], Lotte Verweij RN PhD[1,2], Lauren Clack PhD[1,3], Tracy Finch PhD[4], Marco Riguzzi PhD[1,2], Rahel Naef RN PhD[1,2]

[1]Institute for Implementation Science in Health Care, Faculty of Medicine, University of Zurich

[2]Center of Clinical Nursing Science, University Hospital Zurich

[3]Division of Infectious Diseases and Hospital Epidemiology, University of Zurich, University Hospital of Zurich, Zurich, Switzerland

[4]Department of Nursing, Midwifery & Health, Faculty of Health and Life Sciences, North Umbria University, Newcastle upon Tyne

Corresponding Author:

Saskia Oesch

University of Zürich

Institute for Implementation Science in Health Care

Faculty of Medicine

Universitätstrasse 84, 8006 Zürich

Switzerland

E-Mail: saskia.oesch2@uzh.ch

Phone: +41 79 673 90 87

For peer review only

Abstract

Background

The implementation of complex interventions is considered challenging, particularly in multi-site clinical trials and dynamic clinical settings. This study protocol is part of the FICUS hybrid effectiveness-implementation study. It aims to understand the integration of a multicomponent family support intervention in the real-world context of adult intensive care units. Specifically, the study will assess implementation processes and outcomes of the study intervention, including fidelity, and will enable explanation of the clinical effectiveness outcomes of the trial.

Methods and Analysis

This mixed methods multiple case study is guided by two implementation theories, the Normalization Process Theory and the Consolidated Framework for Implementation Research. Participants are key clinical partners and health care professionals of eight intensive care units allocated to the intervention group of the FICUS trial in the German-speaking part of Switzerland. Data will be collected at four time points over the 18-month active implementation and delivery phase using qualitative (small group interviews, observation, focus group interviews) and quantitative data collection methods (surveys, logs). Descriptive statistics and parametric and non-parametric tests will be used according to data distribution to analyze within and between cluster differences, similarities and factors that influence fidelity and the level of integration over time. Qualitative data will be analyzed using a pragmatic rapid analysis approach and content analysis.

Ethics and dissemination

Ethics approval was obtained from the Cantonal Ethics Committee of Zurich BASEC ID 2021-02300 (08. February 2022). Study findings will provide insights into implementation and its contribution to intervention outcomes, enabling understanding of the usefulness of applied implementation strategies and highlighting main barriers that need to be addressed for scaling the intervention to other health care contexts. Findings will be disseminated in peer-reviewed journals and conferences.

Protocol registration

Open science framework (OSF) https://osf.io/8t2ud Registered on 21 December 2022.

For peer review only

Strengths and limitations

- The use of hybrid study designs to understand intervention and implementation outcomes is key in complex intervention health research.
- The proposed study draws on established implementation science theories and methods to investigate the integration and delivery of a complex family support intervention in a highly dynamic clinical environment.
- A strength of this mixed-methods study is a balanced use of rigorous and pragmatic data collection and analysis strategies to inform ongoing implementation while also generating generalizable knowledge on implementation strategies and determinants around complex family support interventions.
- Given the anticipated small sample sizes per case, potential limitations will be a small or incomplete quantitative data set due to staff turnover or low response rate. Another potential limitation of the study might be the data collection burden on participants.
- The use of several data sources and the combination of qualitative and quantitative data will enable a comprehensive understanding of the phenomenon under study.

For peer review only - http://bmjopen.bmj.com/site/about/guidelines.xhtml

INTRODUCTION

Up to sixty percent of family members of critically ill patients in intensive care units (ICU) experience high levels of stress and uncertainty, which may lead to negative mental health outcomes (1-3). Families require consistent involvement, communication, and support, however, there is a lack of research on the clinical effectiveness and successful implementation of family-focused care in ICUs (3). To better support family health and to close this know-do gap in the area of family-focused ICU care, the FICUS study was launched to investigate the effectiveness and implementation of a nurse-led, interprofessional delivered family support intervention (FSI) in Swiss ICUs (4). Successful implementation of the three FSI components, namely family engagement, support, and communication by a family nurse and interprofessional team along the patient pathway is expected to increase the likelihood of clinical effectiveness, and will be critically influenced by the context into which the intervention is introduced, particularly as part of a multisite clinical trial (5-8).

Specific implementation guidance for integrating FSIs in critical health care settings is still scarce. Previously published research focusing on family nursing implementation have mainly focused on promoting nurses' skills development and open attitudes toward family-focused care (9-13). Main barriers to implement family-focused care in ICUs have been described as lack of understanding of what needs to be done to achieve family-centered care (e.g., lack of support from fellow nurses), organizational barriers (e.g., daily workload), individual barriers (e.g., health care professionals' attitudes), and barriers related to interprofessional care (e.g., communication barriers). In contrast, leadership support, clear and accurate communication, and engagement of the health care team are described as the most important facilitators (14-24). However, intervention research around FSIs in ICUs has not investigated their implementation (12, 25, 26), except for one ongoing trial, which will explore implementation processes and outcomes (27).

The implementation of complex interventions, such as FSIs, is challenging as they contain several independently and interdependently interacting components at the individual behavioral, interprofessional team, and organizational level (6-8, 28-30). Implementation of new interventions involves changes at the individual behavioral, team and organizational level (31) and several contextual determinants that influence successful integration may appear (32). A growing body of research focuses on the development of theories and frameworks to optimize the design and implementation of complex interventions (7, 8, 33, 34), but implementation success remains still widely variable (35). FSIs pose greater challenges for implementation evaluation and cannot rely on preestablished cause and effect specifications (36). As recommended within implementation science and complex intervention research, the focus should not only lie on the clinical effectiveness of a given intervention, but on the conditions needed to achieve the intervention's reach and impact in the real-world context (7, 8). Randomized controlled trials are important in establishing intervention effectiveness, however they focus solely on

For peer review only

clinical health or service outcomes (37, 38). Even if implementation outcomes, such as fidelity, serve as intermediate outcomes in studies of intervention effectiveness (39), in-depth exploration of implementation, particularly in the field of FSIs, has received scant attention (12, 40).

A focus on implementation is needed to learn about successful integration processes of FSIs in critical care settings. This paper reports an implementation study protocol for investigating the implementation process and outcomes of a multicomponent FSI alongside a cluster-randomized clinical trial (FICUS) (4), which examines the clinical effectiveness.

METHODS

Design

The current study is part of the FICUS hybrid effectiveness-implementation study. To investigate the implementation processes and outcomes of a complex phenomenon, that is the FSI in its “real-world” context (41), we will use an embedded multiple case study - mixed methods convergent design (42, 43)**,** in which each of the participating ICUs is conceptualized as a “case” (44). Two research questions will be investigated:

1. What influence does the integration process of the FSI in eight ICUs have on the level of integration, mechanisms of impact and the implementation outcomes defined as fidelity, feasibility, acceptability, appropriateness, and sustainability?
2. Which contextual determinants (barriers and facilitators) influence the integration process and explain its impact on the intervention outcomes?

Study overview

Qualitative and quantitative data will be collected at four timepoints Figure 1. First, data will be collected as part of a formative process evaluation during the adaptive implementation process at three timepoints over 12 months (T1-T3). Rapid, primarily qualitative data collection procedures, such as small group interviews, observation and case conferences will be applied (45-47), coupled with structured self-assessment tools, and integrated into a series of meetings and activities with key clinical partners that serve to support implementation and to ensure quality of intervention delivery. A summative evaluation will be held with key clinical partners and ICU staff after the conclusion of the implementation / delivery phase (T4) to assess all study endpoints, using quantitative and qualitative assessment methods, such as focus group interviews and surveys.

Figure 1 Implementation study overview

Guiding conceptual frameworks and theories

The Normalization Process Theory (NPT) (48, 49) and the Consolidated Framework for Implementation Research (CFIR) (50) will guide the evaluation of the implementation. While NPT explains changes in the

For peer review only

For peer review only - http://bmjopen.bmj.com/site/about/guidelines.xhtml

way people think about and act to integrate a new intervention, CFIR categorizes and describes contextual determinants that influence implementation (51). NPT and CFIR will be combined and integrated in all stages of the research process, including conceptualization, data collection, analysis, and dissemination. Table 1 provides an overview and description of NPT and CFIR in the implementation study.

We developed an Implementation Research Logic Model (IRLM) drawing on CFIR (50), NPT (31), and Proctor's Conceptual Framework for Implementation Research (39) to develop a specific implementation theory around the FSI that outlines contextual determinants (52) implementation mechanisms of impact, and outcomes Figure 2 .

Table 1 Selected implementation science frameworks and theories used within the FICUS Implementation Study

**Framework / Theory**	**Type and description**	**Relevance to the FICUS Implementation Study**
**NPT**	The NPT is a middle-range sociological theory that conceptualizes implementation, embedding and integration of complex interventions in health care settings (49). It focuses on the work required to embed and normalize an intervention in routine practice. NPT consists of 4 key constructs (coherence, cognitive participation, collective action & reflexive monitoring) and 16 sub-constructs (53).	NPT provides a flexible framework to evaluate integration and embedding of the FSI. NPT will help to first, better understand the way implementation processes and context shape each other, and second, to explore the collaborative work people do to make sense of the FSI and to integrate it in ICU clinicians' daily routine (54). NPT will be used to investigate and to describe the integration and normalization of the FSI in ICU teams.
**CFIR[1]**	The CFIR is a pragmatic meta-theoretical determinant framework which provides a pragmatic structure to guide process evaluations. CFIR provides an overarching typology to promote implementation theory development and verification about what works where and why across multiple contexts (50). It includes five domains (inner setting, outer setting, intervention characteristics, characteristics of individuals involved and processes of implementation) (50). Within the five domains are 37 constructs that can each act as a barrier and / or facilitator to the implementation of an intervention.	The CFIR framework includes a wide range of constructs that relate not only to individual characteristics, but also to structural characteristics of the health care system, as well as on characteristics of the intervention itself. CFIR provides a degree of flexibility where constructs can be selected that are the most relevant to understand individual and system barriers and facilitators associated with the implementation (50) and to assess key contextual determinants to consider within the outer setting and the intervention characteristics domains, which are outside of the NPT scope (51). CFIR will be used to describe potential influencing determinants (barriers and facilitators) during implementation.

For peer review only - http://bmjopen.bmj.com/site/about/guidelines.xhtml

[1] We will use the first version of CFIR (50) because our contextual analysis to adjust the implementation (52) was conducted before the publication of CFIR version 2.0 (55).

143 Figure 2 Implementation Research Logic Model

144 **Setting**

145 The FSI will be implemented in eight of sixteen ICUs participating in the FICUS trial, which have been
146 randomly assigned to the intervention arm (4). All ICUs are certified by the Swiss Society for Intensive
147 Medicine; two are situated in a university-affiliated, and six in a cantonal or teaching hospital.

148 **Participants and sampling**

149 Data will be collected from key clinical partners (KP) and ICU health care professionals (HP). KP are local
150 clinical and management staff involved in the implementation and delivery of the FSI (n=45) and will
151 include local implementers (LI) who support the implementation process, trained family nurses (FN), who
152 are registered nurses with a certification in ICU nursing or equivalent, who are trained in family systems
153 nursing and deliver the FSI, as well as a nurse and physician team leaders and/or their deputy (TL).
154 Quantitative and qualitative data will be gathered from all KP during the process evaluation.
155 The second participant group will include HP working on the participating ICUs (approximate n ≈ 750).
156 Quantitative data for the summative evaluation will be gathered from all HP with an anticipated response
157 rate of 50-70% using a comprehensive sampling strategy. To increase response rates, 3-weekly reminder
158 e-mails will be sent twice. Qualitative data will be gathered from a subsample of HP (n= 80-100) using a
159 purposive sampling strategy. Inclusion criteria will be: ICU physicians or nurses working clinically on study
160 units, having an active or influential role in the implementation / delivery of the FSI and / or having either
161 knowledge / experience with the FSI and / or a clinical leadership responsibility, having worked at the ICU
162 for at least 6 months.

163 **Study endpoints**

164 The study endpoints pertain to three conceptual dimensions: 1) process indicators, 2) implementation
165 outcomes and 3) contextual determinants. Table 2 provides a summary of the conceptual definitions, data
166 sources, and measurement.

167 *Process indicators*

168 The implementation process and integration mechanisms will be investigated to gain an in-depth
169 understanding of the implementation from the perspective of KP. It is of particular interest how the FSI
170 becomes (or fails to become) integrated into interprofessional care delivery to families and a normalized
171 ICU care process (48). Indicators will include self-perceived integration of the intervention in the
172 interprofessional ICU team and the overall level of integration in ICU processes.

For peer review only

Implementation outcomes

We will investigate the implementation outcomes of fidelity, feasibility, acceptability, appropriateness and sustainability (39). As fidelity is vital to evaluate our intervention and implementation theory for predicting effectiveness outcomes (56, 57), we chose it as the primary outcome. Fidelity is defined according to the Conceptual Framework for Implementation Fidelity (58, 59), and has been operationalized within the three domains of delivery, receipt, and enactment as outlines by Bellg et al. (60).

We will investigate feasibility, appropriateness, and acceptability because these outcomes play a critical role in implementation research and serve as indicators of the effect of the implementation process and as prerequisites for achieving the desired service and family outcomes (61). To understand how the implementation develops over time and how to best support long-term delivery, we will investigate sustainability as well as post- implementation sustainment outcomes (62).

Contextual determinants

Contextual determinants at the level of individuals, the team, the organization, and the implementation itself, namely barriers and facilitators that hinder or support successful implementation, will be assessed from a clinician’s perspective guided by the CFIR (50) and NPT (48).

For peer review only

Table 2 Study endpoints, definition, data source operationalization and data collection timepoints

STUDY ENDPOINTS	DEFINITION	DATA SOURCE	OPERATIONALIZATION	DATA COLLECTION TIMEPOINT T1	T2	T3	T4
**<u>Implementation Process</u>**							
Process indicators	Course of integration of FSI in team / care delivery processes and observed integration mechanisms	KP	Small group interviews[1]	x		x	
		KP, HP	NoMAD [1, 2, 3]			x	x
		KP	Implementation rating tool [1]	x		x	
**<u>Implementation Outcomes</u>**							
**Fidelity: Delivery**							
Relative frequency	Number of interventions per length of stay (LoS)	-	Intervention fidelity log[1,9]	x	x	x	x
Absolute dose	Total minutes of interventions	-	Intervention fidelity log[1,9]	x	x	x	x
Consistency	Consistent delivery of the FSI within and across ICUs as defined per intervention manual	-	Intervention fidelity log[1,9]	x	x	x	x
Availability	Availability of FN (days) as defined per trial protocol to ensure intervention capacity	KP	Intervention capacity assessment[1]	x		x	
		FN	Focus group interviews[2]				x
**Fidelity: Receipt**							
Attendance at case conferences	Overall participation rate	FN, (LI)	Case conferences[1,4]	x	x	x	x
Comprehension & skills	Skills in working systematically with families	FN	FNPS[1, 5]	x	x		x
		FN	Focus group interviews[2]				x
**Fidelity Enactment**							
Engagement & performance	Quality and consistency of intervention performance	FN	Fidelity Self-Rating Tool[1]		x		x
		Researcher, FN	Semi-structured observation[1]		x		
		FN	Focus group interviews[2]				x
**Feasibility**	Extent to which the FSI can be successfully used and carried out within ICU settings	KP	FIM[1,2,6]	x		x	x
		KP, HP	Focus group interviews[2]				x
**Acceptability**	Perception among clinical partners that the FSI is agreeable	KP	IAM[1,2,7]	x		x	x
		KP, HP	Focus group interviews				x
**Appropriateness**	Perceived fit, relevance and compatibility of the FSI for clinical partners	KP, HP	AIM[1,2,8]	x		x	x
		KP, HP	Focus group interviews[2]				x
**Sustainability / Sustainment**	Existence of FN role assessed 6 and 12 months after study end	KP	Sustainability implementation survey[10]				x
	How likely the FSI is to be sustained in the future with necessary structures and processes in place to maintain FSI in ICUs	KP; HP	Focus group interviews[2]				x

For peer review only

STUDY ENDPOINTS	DEFINITION	DATA SOURCE	OPERATIONALIZATION	DATA COLLECTION TIMEPOINT T1	T2	T3	T4
**Contextual Determinants**	Influencing barriers and facilitators as conceptualized in CFIR and NPT	KP	Small group interviews[1]	x		x	
		KP, HP	Focus group interviews[2]				x

Abbreviations: FN Family Nurses, LI local implementers, KP Key clinical partners, HP Health Care Professionals, ICU Intensive Care Unit, LoS Length of stay, CFIR Consolidated Framework for Implementation Research, NPT Normalization Process Theory

[1] Part of the process evaluation
[2] Part of the summative evaluation
[3] German Version of the Normalization Measure Development Questionnaire (NoMAD) (63)
[4] Monthly case conferences throughout the 18-months active implementation and delivery phase, attendance is mandatory for FN, voluntary for LI
[5] German version of the Family Nursing Practice Scale (FNPS) (64)
[6] German version of the Feasibility Intervention Measure (FIM) (65)
[7] German version of the Intervention Appropriateness Measure (65)
[8] German version of the Acceptability Intervention Measure (AIM) (65)
[9] Case report forms embedded in RED Cap (66) and completed throughout the study by family nurses / interventionists
[10] Data collection occurs 6 and 12 months after active implementation and intervention delivery / FICUS study end

For peer review only

For peer review only - http://bmjopen.bmj.com/site/about/guidelines.xhtml

Qualitative data collection

Small group interview

Semi-structured small group interviews will be held with KP as part of the process evaluation to assess contextual determinants (barriers and facilitators) and processes that influence the implementation. An interview guide, which is based on NPT and CFIR will be used to explore participants' understanding and perceptions of the intervention delivery, their team progress to achieve integration, and the barriers and facilitators they currently face (54). All interviews will be held face-to-face by two researchers at the respective ICU, audio-recorded, and expected to last 60-90 minutes. Immediately after each interview, key topics will be summarized in a protocol and provided to the KP for validation. This form of member checking will give them the ability to correct errors and wrong interpretations and to create trustworthiness (43) .

Focus group interview

A total of ten focus group interviews will be held as part of the summative evaluation with KP and HP as it is a useful way of collecting qualitative data as social interactions, shared values, cultures and practices which are of particular interest (67).

First, one focus group interview will be held on each ICU with a purposive sample of 8-10 KS and HP, which represent different professional groups, roles, and levels of involvement in the intervention and implementation. These interviews will focus on social and team processes and contextual influences towards the integration and delivery of the FSI.

In addition, two focus group interviews will be held with 8-10 family nurses each to investigate their experiences and perspectives on their role adoption, team-based intervention delivery and clinical work with families, experiences with implementation in the study context, observed benefits to self, team, and families, as well factors that promoted or hindered integration into ICU care delivery.

All focus group interviews will be held face-to-face by two researchers and will take place on site at the respective ICU or an accessible location, in a quiet room to avoid interruptions. Interviews will be digitally recorded, transcribed, and field notes will be taken. The interviews and the field notes will be transcribed, checked for accuracy, anonymized, and entered into MAXQDA for analysis.

Observation of intervention delivery

A total of eight semi-structured observations of intervention delivery by trained FN, that is, one per ICU, will be conducted to assess intervention fidelity and the establishment of structures for the FSI using a semi-structured fidelity audit observation tool. The tool bases on the intervention manual and was developed by the research team. It consists of four items on available structures for the FICUS study and 59 FSI activities corresponding to the three main components 1) engaging and liaising, 2) supporting and 3) communicating. Activities will be rated according to their performance by the FN (performed, partially performed, not performed, not applicable). In addition, the timepoint

For peer review only - http://bmjopen.bmj.com/site/about/guidelines.xhtml

of the conversation (e.g., admission) and the type of conversation (e.g., on-site, via telephone) will be documented. An additional text box at the end of the tool will allow to document observations about the ICU context. The tool was pretested once by one researcher. All observations will be conducted by a doctoral student or a researcher, which are both trained in family systems nursing and are expected to last 2-4 hours each.

Case conference

During monthly case conferences, field notes will be taken by a doctoral student to capture FNs' participation rate which is an indicator for fidelity receipt. Participation of the FN will be documented in a structured participation form. All case conferences will be held online by two senior researchers trained in family systems nursing and will last 60 minutes.

Quantitative measures

Fidelity Self-Rating Tool (Fidelity-SRT)

To capture quality and consistency of intervention performance (fidelity enactment), FN's appraisal of self-perceived engagement and performance in FSI delivery will be assessed two times using the Fidelity-SRT. The tool is based on the intervention manual and was developed by the FICUS research team. It includes 3 subscales according to the intervention components: 1) engaging and liaising (15 items), 2) supporting (27 items) and 3) communicating (15 items). All items will be rated on 5-https://osf.io/czjt8/point Likert scale (1 = never to 5 = always) regarding the last 3-5 families taken care of by the FN. A low mean score indicates low self-perceived success in FSI performance whereas a higher score means higher succeed in intervention delivery. The Fidelity-SRT was pretested by two KP and requires approximately 10 minutes to complete.

Intervention Capacity Assessment (ICA)

To assess FN's availability as defined per trial protocol to ensure provision of intervention capacity (fidelity delivery), the ICA will be completed two times during process evaluation by KP's. The ICA consists of two self-developed items referring to the four weeks prior to the assessment and include: 1) Was there always at least one FN available to deliver the FSI during five days per week? 2) (only for FN) To what extent was it possible to provide the FSI to family members according to the timeline requirements of the protocol and the needs of the family? Both items will be rated on a 5-point Likert scale ranging from 0 (never) to 5 (I don't' know).

Intervention Fidelity Logs (IFL)

FSI characteristics, such as duration in minutes, frequency, delivery mode, intervention activity (as defined in the intervention manual) will be recorded in a structured, online intervention log, which is part of the clinical trial electronic data capture system REDCap (66). The logs are completed by FN for each included trial participant on an ongoing basis.

For peer review only - http://bmjopen.bmj.com/site/about/guidelines.xhtml

Family Nursing Practice Scale (FNPS)

To assess FN's appraisal of practice skills and their reflection in working with families, the 10-item German version of the "Family Nursing Practice Scale" will be used (64). Items will be scored on a 5-point Likert-type scale ranging from one (high level) to five (low level). Lower mean scores will represent higher practice skills. The FNPS was validated in critical care, suggesting high internal consistency (Crohnbach's alpha 0.84) (64)

Feasibility Intervention Measure (FIM), Acceptability of Intervention Measure (AIM) and Intervention Appropriateness Measure (IAM)

To assess KPs' perceived level of feasibility, acceptability, and appropriateness of the FSI, adapted psychometrically validated German versions of the FIM, AIM and IAM will be completed three times. Each of these surveys include four items that utilize a 5-point Likert scale ranging from one (completely disagree) to five (completely agree). A recurring adaption is made by inserting "FSI" into the appropriate space on the instrument e.g., "(Insert Intervention) is doable" will become "FSI is doable". All three surveys are reliable and pragmatic instruments with a high Crohnbach's alpha (FIM α = .89; AIM α = .80, IAM; α= 0.87) (61, 65).

Normalization Measure Development questionnaire (NoMAD)

To assess HP's perceived level of integration, the German version of the NoMAD will be used two times (63). It includes 20 items utilizing a 5-point Likert scale ranging from one (completely disagree) to five (completely agree) and is adaptable by specifying the "(the intervention)" to study purpose (68), that is, the "FSI". The NoMAD instrument has high face validity, construct validity and internal consistency for assessing perceptions of factors relevant to the integration of the FSI. Internal consistency (Crohnachs's alpha) was reported as follows: coherence (4 items, α=0.71); collective action (7 items, α= 0.78); cognitive participation (4 items, α= 0.81) and reflexive monitoring (5 items, α= 0.65). Overall, the normalization scale was described as highly reliable (20 items, α= 0.89) (68).

Sustainability Implementation Survey

To assess both, KP's appraisal of sustainability determinants and post- implementation sustainment outcomes a self-developed sustainability survey will be sent at six and twelve months after the last intervention delivery and before the follow-up completion by the last study participant. The survey will include questions about the extent to which the FSI is still delivered as intended after FICUS study support is terminated (sustainability) and continued enactment of processes and practices in daily ICU routine that have been conveyed and learned through the FSI (69). All items will be rated on a 5-point Likert scale ranging from one (completely disagree) to completely agree).

Demographics

Information on participants' age, gender, profession, institution, level of employment, level of education, work experience in the ICU, overall work experience in the profession and whether family

For peer review only

For peer review only - http://bmjopen.bmj.com/site/about/guidelines.xhtml

nursing was part of the curriculum of their training and/or studies, will be collected. Furthermore, cluster data at ICU level will be collected to describe the eight cases in detail. Cluster data will include ICU cluster size (number of health care professionals), number of beds, number of admissions per year, and average length of stay.

All surveys will be implemented in UNIPARK software.

Data analysis

Qualitative data analysis

Data analysis of the process evaluation will require prompt analysis of interview data to rapidly develop or modify implementation strategies in case of need. Hence, small group interviews will be analyzed using an adapted approach of Rapid Identification of Themes from Audio recordings (RITA) (70), in combination with a deductive approach guided by CFIR / NPT (47). This form of directed content analysis will apply the following steps: 1) specifying the evaluation foci, 2) identifying themes from audio recordings, 3) creating a codebook, 4) coding and refining the codebook and 5) sorting codes into themes inductively and deductively drawing on CFIR and NPT constructs (71).

Qualitative data from the summative evaluation (T4) will be analyzed using content analysis, which allows the use of deductive and inductive strategies. It uses the following steps: 1) reading each of the transcribed interviews to get familiar with the data, taking notes of first impressions to gain a sense of the whole phenomenon, 2) identifying and developing codes for each unit of meaning, 3) developing of definitions for each code and group them into categories of related meanings, and 4) synthesizing codes and categories into themes (71, 72).

Quantitative data analysis

Raw data will be verified, encrypted, and entered into R Project for analysis. Descriptive statistics will be computed for the entire sample, by case and by data collection timepoint. Depending on scale level and distribution, results will be presented either as absolute and relative frequencies, as mean and standard deviation, or as median and interquartile range. To compare differences in central tendency between clusters, data will be analyzed using parametric or non-parametric inferential statistical tests for paired or unpaired data. For comparisons of nominal data between clusters or individuals (unpaired), a chi-squared test will be used if observed and expected frequencies allow for it, and Fisher's exact test will be used otherwise. To detect within-case changes across timepoints, paired non-parametric tests of proportions and central tendency will be used (possibly extensions for unbalanced data) (73).

Mixed-methods and cross-case analysis

To provide an in-depth understanding of the phenomenon under study, and to increase the validity of our findings, data triangulation will occur on case level by comparing qualitative and quantitative data sets (44, 74). Data comparison occurs after the separate analysis of qualitative and quantitative

For peer review only - http://bmjopen.bmj.com/site/about/guidelines.xhtml

data (43, 75, 76). Datasets will be merged by listing and joining results on a same table, summarizing raw data that are important to consider and displaying statistics and themes of each case in a matrix. Qualitative data will be mixed with quantitative data in relation to the implementation outcomes (i.e., qualitative data on acceptability with IAM data) and the implementation process (i.e., qualitative data on integration with NoMAD data) using comparing, contrasting, and constantly verifying methods (76).

Integration will also occur through construction of cases to develop a robust case presentation (42, 74). We will compare-contrast cases to not only identify patterns within but also across cases to build explanations for a successful implementation. To compare different cases, cross-case synthesis will be used, to compare and to identify similarities and differences between the cases. This will involve tables that display the data according to a uniform framework to compare across cases for both research questions and site-specific experiences to emerge from the data (44). Finally, data will be brought together in a logic model including moderating influencers (77).

Data management

Intervention log data is stored on a MySQL database server hosted by the Clinical Trials Unit Zurich, which holds a REDCap End-User License Agreement for this electronic data capture system (4). Unencrypted primary data, such as fieldnotes and audiotapes, will be stored in a lockable compartment to which only the research team has access. All documents are kept encrypted on the University of Zurich server and the key will be destroyed after completion of the study. Essential documents will be retained for at least 10 years after termination of the study.

CONTRIBUTION TO THE LITERATURE AND DISSEMINATION

This protocol outlines the rationale, design and methods for a process and outcome evaluation of a new, multicomponent, nurse-led FSI in ICUs. The key feature of the study design is the development of flexible and pragmatic data collection methods to capture data across five areas: 1) if the FSI was delivered as intended (fidelity), 2) how clinicians experienced the implementation process of the FSI , 3) which determinants (barriers and facilitators) influenced the integration process, 4) the impact of tailored implementation strategies to overcome contextual determinants and 5) to explain / interpret the clinical effectiveness results. Findings will be used to build a comprehensive understanding of how and why the FSI in ICUs was effectively implemented and delivered, and to delineate recommendations for scale-up to further ICUs and / or adaptions to different clinical contexts. Study findings will make a significant contribution to the current body of knowledge in attending to implementation of FSIs, as there is only currently one study protocol by Curtis et al. (27) outlining plans to explore implementation. This study protocol may serve as a useful guidance for planning similar studies and projects that aim to test and / or implement complex interventions in real-world contexts, such as a nurse-led family support program in ICUs.

For peer review only

For peer review only - http://bmjopen.bmj.com/site/about/guidelines.xhtml

Our findings will be disseminated to all partners and stakeholders involved and interested in its long-term implementation. Research findings will be disseminated via peer-reviewed journals and conferences.

ETHICS

The FICUS study, including the embedded implementation study has been approved by the responsible Swiss cantonal ethics committees (Nr. 2021-02300). Study participants will be informed about the purpose of this study and study participation orally and received a study information pack. Relevant national and international data protection regulation will be respected in accordance with the principles of the Declaration of Helsinki (78).

Abbreviations

AIM: Acceptability Intervention Measure
CC: Case Conferences
CFIR: Consolidated Framework for Implementation Research
FICUS: Family Intensive Care Units
Fidelity-SRT: Fidelity Self Rating Tool
FIM: Feasibility Intervention Measure
FN: Family Nurses
FNPS: Family Nursing Practice Scale
FSI: Family Support Intervention
HP: Health Care Professionals
IAM: Intervention Appropriateness Measure
ICA: Intervention Capacity Assessment
ICU: Intensive Care Unit
IFL: Intervention Fidelity Logs
KP: Key Clinical Partners
LI: Local Implementers
NoMAD: Normalization Measure Development Questionnaire
NPT: Normalization Process Theory
OSF: Open Science Framework
PICS-F: Post-Intensive Care Syndrome-Family
RITA: Rapid Identification of Themes from Audio recordings
TL: Team Leaders

Consent for publication

Not applicable.

Availability of data and materials

Data sharing is not applicable to this article as no datasets were generated or analyzed during the current study (study protocol).

Competing interests

The authors declare that they have no competing interests.

For peer review only

For peer review only - http://bmjopen.bmj.com/site/about/guidelines.xhtml

Funding

The study is funded by the Swiss National Science Foundation (SNSF, No. 33IC30_198778/1). The funder has no role in the development of the study design; collection, analysis, or interpretation of the data; writing of the manuscript; or decision to submit the manuscript for publication.

Authors' contributions

SO, LV and RN conceptualized the study. The manuscript was written by SO and LV. RN provided ongoing feedback and critically revised the manuscript. MR supported the designing of the statistical analysis plan. TF and LC critically reviewed the study design and manuscript for important intellectual content. All authors have reviewed the drafts and approved the final version.

Acknowledgements

We are very grateful to the FICUS study group for supporting this study.

References

1. Davidson JE, Aslakson RA, Long AC, Puntillo KA, Kross EK, Hart J, et al. Guidelines for Family-Centered Care in the Neonatal, Pediatric, and Adult ICU. Critical care medicine. 2017;45(1):103-28.
2. Gerritsen RT, Hartog CS, Curtis JR. New developments in the provision of family-centered care in the intensive care unit. Intensive Care Medicine. 2017;43(4):550-3.
3. Kiwanuka F, Sak-Dankosky N, Alemayehu YH, Nanyonga RC, Kvist T. The evidence base of nurse-led family interventions for improving family outcomes in adult critical care settings: A mixed method systematic review. International Journal of Nursing Studies. 2022;125:104100.
4. Naef R, Filipovic M, Jeitziner M-M, von Felten S, Safford J, Riguzzi M, et al. A multicomponent family support intervention in intensive care units: study protocol for a multicenter cluster-randomized trial (FICUS Trial). Trials. 2022;23(1):533.
5. May C, Finch T, Mair F, Ballini L, Dowrick C, Eccles M, et al. Understanding the implementation of complex interventions in health care: the normalization process model. BMC Health Services Research. 2007;7(1):148.
6. Moore GF, Audrey S, Barker M, Bond L, Bonell C, Hardeman W, et al. Process evaluation of complex interventions: Medical Research Council guidance. BMJ : British Medical Journal. 2015;350:h1258.
7. Pfadenhauer LM, Gerhardus A, Mozygemba K, Lysdahl KB, Booth A, Hofmann B, et al. Making sense of complexity in context and implementation: the Context and Implementation of Complex Interventions (CICI) framework. Implementation Science. 2017;12(1):21.
8. Skivington K, Matthews L, Simpson SA, Craig P, Baird J, Blazeby JM, et al. A new framework for developing and evaluating complex interventions: update of Medical Research Council guidance. BMJ. 2021;374:n2061.
9. Duhamel F, Dupuis F, Turcotte A, Martinez AM, Goudreau J. Integrating the Illness Beliefs Model in clinical practice: a Family Systems Nursing knowledge utilization model. Journal of Family Nursing. 2015;21(2):322-48.
10. Goldfarb M, Bibas L, Burns K. Patient and Family Engagement in Care in the Cardiac Intensive Care Unit. Canadian Journal of Cardiology. 2020;36(7):1032-40.
11. Naef R, Kaeppeli BM, Lanter R, Petry H. Implementing Family Systems Care Through an Educational Intervention With Nurses and Midwives in Obstetrics and Gynecological Care: A Mixed-Methods Evaluation. Journal of Family Nursing. 2020;26(2):138-52.
12. White DB, Angus DC, Shields AM, Buddadhumaruk P, Pidro C, Paner C, et al. A Randomized Trial of a Family-Support Intervention in Intensive Care Units. The New England Journal of Medicine. 2018;378(25):2365-75.

For peer review only

For peer review only - http://bmjopen.bmj.com/site/about/guidelines.xhtml

13. Svavarsdottir EK, Sigurdardottir AO, Konradsdottir E, Stefansdottir A, Sveinbjarnardottir EK, Ketilsdottir A, et al. The process of translating family nursing knowledge into clinical practice. Journal of Nursing Scholarship. 2015;47(1):5-15.

14. Kiwanuka F, Shayan SJ, Tolulope AA. Barriers to patient and family-centred care in adult intensive care units: A systematic review. Nurs Open. 2019;6(3):676-84.

15. Hamilton R, Kleinpell R, Lipman J, Davidson JE. International facilitators and barriers to family engagement in the ICU: Results of a qualitative analysis. Journal of Critical Care. 2020;58:72-7.

16. Price AM, McAndrew NS, Thaqi Q, Kirk M, Brysiewicz P, Eggenberger S, et al. Factors influencing critical care nurses' family engagement practices: An international perspective. Nursing in Critical Care. 2022;n/a(n/a).

17. Naef R, Brysiewicz P, Mc Andrew NS, Beierwaltes P, Chiang V, Clisbee D, et al. Intensive care nurse-family engagement from a global perspective: A qualitative multi-site exploration. Intensive and Critical Care Nursing. 2021;66:103081.

18. Imanipour M, Kiwanuka F. Family nursing practice and family importance in care – Attitudes of nurses working in intensive care units. International Journal of Africa Nursing Sciences. 2020;13:100265.

19. Esmaeili M, Cheraghi MA, Salsali M. Explaining the Context of Patient-Centered Care in Critical Care Units. Journal of Holistic Nursing and Midwifery. 2017;27(3):17-26.

20. Thirsk LM, Vandall-Walker V, Rasiah J, Keyko K. A Taxonomy of Supports and Barriers to Family-Centered Adult Critical Care: A Qualitative Descriptive Study. Journal of Family Nursing. 2021;27(3):199-211.

21. Hetland B, Hickman R, McAndrew N, Daly B. Factors Influencing Active Family Engagement in Care Among Critical Care Nurses. AACN Advanced Critical Care. 2017;28(2):160-70.

22. Kleinpell R, Zimmerman J, Vermoch KL, Harmon LA, Vondracek H, Hamilton R, et al. Promoting Family Engagement in the ICU: Experience From a National Collaborative of 63 ICUs. Critical Care Medicine. 2019;47(12):1692-8.

23. Burns KEA, McDonald E, Debigaré S, Zamir N, Vasquez M, Piche-Ayotte M, et al. Patient and family engagement in patient care and research in Canadian intensive care units: a national survey. Canadian Journal of Anesthesia/Journal canadien d'anesthésie. 2022;69(12):1527-36.

24. Thürlimann E, Verweij L, Naef R. The Implementation of Evidence-Informed Family Nursing Practices: A Scoping Review of Strategies, Contextual Determinants, and Outcomes. Journal of Family Nursing. 2022:10748407221099655.

25. Curtis R, Treece PD, Nielsen EL, Gold J, Ciechanowski PS, Shannon SE, et al. Randomized Trial of Communication Facilitators to Reduce Family Distress and Intensity of End-of-Life Care. American Journal of Respiratory and Critical Care Medicine. 2016;193(2):154-62.

26. Kentish-Barnes N, Chevret S, Valade S, Jaber S, Kerhuel L, Guisset O, et al. A three-step support strategy for relatives of patients dying in the intensive care unit: a cluster randomised trial. Lancet. 2022;399(10325):656-64.

27. Curtis JR, Kentish-Barnes N, Brumback LC, Nielsen EL, Pollak KI, Treece PD, et al. Facilitating communication for critically ill patients and their family members: Study protocol for two randomized trials implemented in the U.S. and France. Contemporary clinical trials. 2021;107:106465.

28. Campbell M, Fitzpatrick R, Haines A, Kinmonth AL, Sandercock P, Spiegelhalter D, et al. Framework for design and evaluation of complex interventions to improve health. BMJ. 2000;321(7262):694-6.

29. Nilsen P, Bernhardsson S. Context matters in implementation science: a scoping review of determinant frameworks that describe contextual determinants for implementation outcomes. BMC Health Services Research. 2019;19(1):189.

30. May CR, Johnson M, Finch T. Implementation, context and complexity. Implementation Science. 2016;11(1):141.

31. May CR, Mair F, Finch T, MacFarlane A, Dowrick C, Treweek S, et al. Development of a theory of implementation and integration: Normalization Process Theory. Implementation Science. 2009;4(1):29.

For peer review only

For peer review only - http://bmjopen.bmj.com/site/about/guidelines.xhtml

32. Groot Kormelinck CM, Janus SIM, Smalbrugge M, Gerritsen DL, Zuidema SU. Systematic review on barriers and facilitators of complex interventions for residents with dementia in long-term care. International Psychogeriatrics. 2021;33(9):873-89.

33. Michie S, Johnston M, Abraham C, Lawton R, Parker D, Walker A. Making psychological theory useful for implementing evidence based practice: a consensus approach. Quality & Safety In Health Care. 2005;14(1):26-33.

34. Nilsen P. Making sense of implementation theories, models and frameworks. Implementation Science. 2015;10(1):53.

35. Presseau J, Grimshaw JM, Tetroe JM, Eccles MP, Francis JJ, Godin G, et al. A theory-based process evaluation alongside a randomised controlled trial of printed educational messages to increase primary care physicians' prescription of thiazide diuretics for hypertension [ISRCTN72772651]. Implementation Science. 2016;11(1):121.

36. Kainz K, Metz A, Yazejian N. Tools for Evaluating the Implementation of Complex Education Interventions. American Journal of Evaluation. 2021;42(3):399-414.

37. Moore GF, Evans RE. What theory, for whom and in which context? Reflections on the application of theory in the development and evaluation of complex population health interventions. SSM - Population Health. 2017;3:132-5.

38. Saunders RP, Evans MH, Joshi P. Developing a process-evaluation plan for assessing health promotion program implementation: a how-to guide. Health Promotion Practice. 2005;6(2):134-47.

39. Proctor E, Silmere H, Raghavan R, Hovmand P, Aarons G, Bunger A, et al. Outcomes for implementation research: conceptual distinctions, measurement challenges, and research agenda. Administration and Policy in Mental Health. 2011;38(2):65-76.

40. Kentish-Barnes N, Chaize M, Seegers V, Legriel S, Cariou A, Jaber S, et al. Complicated grief after death of a relative in the intensive care unit. European Respiratory Journal. 2015;45(5):1341-52.

41. Paparini S, Green J, Papoutsi C, Murdoch J, Petticrew M, Greenhalgh T, et al. Case study research for better evaluations of complex interventions: rationale and challenges. BMC Medicine. 2020;18(1):301.

42. Guetterman TC, Fetters MD. Two Methodological Approaches to the Integration of Mixed Methods and Case Study Designs: A Systematic Review. American Behavioral Scientist. 2018;62(7):900-18.

43. Creswell JW. A Concise Introduction to Mixed Methods Research. 2 ed: SAGE Publications, Inc; 2021.

44. Yin RK. Case Study Research and Applications. COSMOS Corporation; 2018. 319 p.

45. Vindrola-Padros C, Vindrola-Padros B. Quick and dirty? A systematic review of the use of rapid ethnographies in healthcare organisation and delivery. BMJ Quality & Safety Journal. 2018;27(4):321-30.

46. Palinkas LA, Zatzick D. Rapid Assessment Procedure Informed Clinical Ethnography (RAPICE) in Pragmatic Clinical Trials of Mental Health Services Implementation: Methods and Applied Case Study. Administration and policy in mental health. 2019;46(2):255-70.

47. Nevedal AL, Reardon CM, Opra Widerquist MA, Jackson GL, Cutrona SL, White BS, et al. Rapid versus traditional qualitative analysis using the Consolidated Framework for Implementation Research (CFIR). Implementation Science. 2021;16(1):67.

48. May CR, Cummings A, Girling M, Bracher M, Mair FS, May CM, et al. Using Normalization Process Theory in feasibility studies and process evaluations of complex healthcare interventions: a systematic review. Implementation Science. 2018;13(1):80.

49. May C, Finch T. Implementing, Embedding, and Integrating Practices: An Outline of Normalization Process Theory. Sociology. 2009;43(3):535-54.

50. Damschroder LJ, Aron DC, Keith RE, Kirsh SR, Alexander JA, Lowery JC. Fostering implementation of health services research findings into practice: a consolidated framework for advancing implementation science. Implementation Science. 2009;4(1):50.

51. Schroeder D, Luig T, Finch TL, Beesoon S, Campbell-Scherer DL. Understanding implementation context and social processes through integrating Normalization Process Theory (NPT) and the Consolidated Framework for Implementation Research (CFIR). Implementation Science Communications. 2022;3(1):13.

For peer review only - http://bmjopen.bmj.com/site/about/guidelines.xhtml

52. Verweij L, Oesch S, Naef R. Tailored Implementation of the FICUS multicomponent family support intervention in adult intensive care units: Findings from a mixed methods contextual analysis [Manuscript submitted for publication]. Implementation Science 2023.

53. Finch TL, Mair FS, O'Donnell C, Murray E, May CR. From theory to 'measurement' in complex interventions: methodological lessons from the development of an e-health normalisation instrument. BMC medical research methodology. 2012;12:69-.

54. May CR, Albers B, Bracher M, Finch TL, Gilbert A, Girling M, et al. Translational framework for implementation evaluation and research: a normalisation process theory coding manual for qualitative research and instrument development. Implementation Science. 2022;17(1):19.

55. Damschroder LJ, Reardon CM, Widerquist MAO, Lowery J. The updated Consolidated Framework for Implementation Research based on user feedback. Implementation Science. 2022;17(1):75.

56. Haynes A, Brennan S, Redman S, Williamson A, Gallego G, Butow P, et al. Figuring out fidelity: a worked example of the methods used to identify, critique and revise the essential elements of a contextualised intervention in health policy agencies. Implementation Science. 2016;11(1):23.

57. Ginsburg LR, Hoben M, Easterbrook A, Anderson RA, Estabrooks CA, Norton PG. Fidelity is not easy! Challenges and guidelines for assessing fidelity in complex interventions. Trials. 2021;22(1):372.

58. Hasson H, Blomberg S, Dunér A. Fidelity and moderating factors in complex interventions: a case study of a continuum of care program for frail elderly people in health and social care. Implementation Science. 2012;7(1):23.

59. Carroll C, Patterson M, Wood S, Booth A, Rick J, Balain S. A conceptual framework for implementation fidelity. Implementation Science. 2007;2(1):40.

60. Bellg, Borrelli B, Resnick B, Hecht J, Minicucci DS, Ory M, et al. Enhancing treatment fidelity in health behavior change studies: best practices and recommendations from the NIH Behavior Change Consortium. Health Psychol. 2004;23(5):443-51.

61. Weiner, Lewis CC, Stanick C, Powell BJ, Dorsey CN, Clary AS, et al. Psychometric assessment of three newly developed implementation outcome measures. Implement Science. 2017;12(1):108.

62. Hall A, Shoesmith A, Doherty E, McEvoy B, Mettert K, Lewis CC, et al. Evaluation of measures of sustainability and sustainability determinants for use in community, public health, and clinical settings: a systematic review. Implementation Science. 2022;17(1):81.

63. ImpleMentAll. Normalization Measure Development Questionnaire (short NoMAD). German Version: ImpleMentAll; 2018 [Available from: https://www.implementall.eu/NoMAD_German.pdf.

64. Naef R, Ernst J, Müeller M, Schmid-Mohler G. Translation and Psychometric Validation of the German Version of the Family Nursing Practice Scale (FNPS). Journal of Family Nursing. 2021;27(1):34-42.

65. Kien, Schultes, Szelag, Schoberberger, Gartlehner. German language questionnaires for assessing implementation constructs and outcomes of psychosocial and health-related interventions: a systematic review. Implementation Science. 2018;13(1):150.

66. https://www.project-redcap.org/. [Available from: https://www.project-redcap.org/.

67. Ritchie J, Lewis J. Qualitative research practice. A Guide for Social Science Students and Researchers. London, Thousand Oaks, New Dehli: SAGE Publications; 2003. 331 p.

68. Finch T, Girling M, May C, Mair FS, Murray E, Treweek S, et al. Improving the normalization of complex interventions: part 2 - validation of the NoMAD instrument for assessing implementation work based on normalization process theory (NPT). BMC Medical Research Methodology. 2018;18(1):135.

69. Chambers DA, Glasgow RE, Stange KC. The dynamic sustainability framework: addressing the paradox of sustainment amid ongoing change. Implementation Science. 2013;8(1):117.

70. Neal JW, Neal ZP, VanDyke E, Kornbluh M. Expediting the Analysis of Qualitative Data in Evaluation: A Procedure for the Rapid Identification of Themes From Audio Recordings (RITA). American Journal of Evaluation. 2014;36(1):118-32.

71. Hsieh HF, Shannon SE. Three approaches to qualitative content analysis. Qualitative Health Research. 2005;15(9):1277-88.

72. Bengtsson M. How to plan and perform a qualitative study using content analysis. NursingPlus Open. 2016;2:8-14.

73. Bland M. An Introducation to Medical Statistics. Oxford: Oxford University Press; 2015.

For peer review only - http://bmjopen.bmj.com/site/about/guidelines.xhtml

609 74. Fetters MD, Molina-Azorin JF. The Journal of Mixed Methods Research Starts a New Decade: The Mixed Methods Research Integration Trilogy and Its Dimensions. Journal of Mixed Methods Research. 2017;11(3):291-307.

612 75. Palinkas LA, Aarons GA, Horwitz S, Chamberlain P, Hurlburt M, Landsverk J. Mixed method designs in implementation research. Administration and Policy in Mental Health 2011;38(1):44-53.

614 76. Zhang W, Creswell J. The use of "mixing" procedure of mixed methods in health services research. Medical Care. 2013;51(8):e51-7.

616 77. Mills T, Shannon R, O'Hara J, Lawton R, Sheard L. Development of a 'real-world' logic model through testing the feasibility of a complex healthcare intervention: the challenge of reconciling scalability and context-sensitivity. Evaluation. 2022;28(1):113-31.

619 78. World Medical Association. WMA declaration of Helsinki- Ethical Principles for medical research involving human subjects. World Medical Association; 2022.

For peer review only

For peer review only - http://bmjopen.bmj.com/site/about/guidelines.xhtml

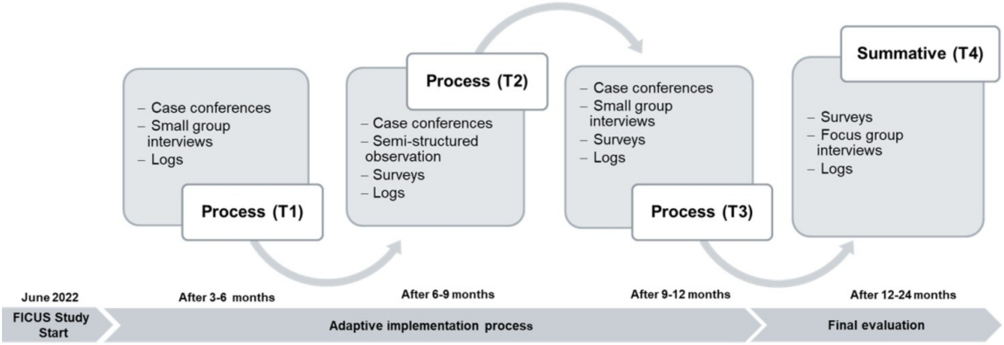

FICUS implementation study overview

250x89mm (150 x 150 DPI)

Determinants[1]

Intervention characteristics

- Integration of the FSI's interprofessional communication component and activities into ICU team processes (+/-)
- Family nurse's co-moderating role during interprofessional family meetings (+/-)

Outer Setting

- FSI delivery depending on recruitment sucess and willingness of family members to participate in study (-)

Inner setting

- FSI training program for family nurses (+)
- ICU culture with a high value placed on family engagement and support (+)
- ICU interprofessional team motivation for change (+)
- FICUS research team support (+)
- Staff capacity (-)
- High workload (-)

Characteristics of individuals

- Self-efficacy in new family nursing role (0)
- Time investments needed for intervention delivery and implementation (-)

Process

- Engagement and committment of leadership persons and key clinical partners to support implementation and FSI delivery (+)

Implementation strategies[2]

1. Evaluative and iterative strategies
Regular meetings with key clinical partners
Ongoing feedback loops

2. Adapt and tailor to context
Context assessment with key clinical partners
Tailored implementation plans

3. Train and educate stakeholders
Family nurse education
Team information meetings
Implementation support person training

4. Provide interactive assistance
ICU leadership support
Local and external implementation support
Nurse and physician champions

5. Develop stakeholder interrelationships
Leadership and key clinical partner's engagement
Local nurse and physician champions

6. Support clinicians
Individual coaching and support
Case conferences

Mechanisms[2]

1. Assure that quality improvement processes are targeted at FSI delivery and integration. Assess progress and ensure mutual reflection on study progress. Enable adaptations to address changing contextual Influencers.

2. Raising awareness about ICU context and its influence on FSI delivery and integration, including factors that support or endanger adoption. Enabling targeted actions and / or collective changes to contextual factors that influence FSI delivery and integration.

3. Providing and ensuring the necessary knowledge, skill, and self-efficacy of ICU team members to deliver the FSI.

4. Assure responsiveness to day-to-day challenges in FSI delivery and integration. Ensure capacity for change in FSI delivery and integration.

5. Capitalize on individual and team motivation and capacity (skills, competencies, attitudes) to improve care and to advance (nursing, collaborative) practice. Create capacity for change in family-focused care through research and implementation. Ensure ownership and sense of community in improving family-focused care

6. Assure consistent delivery of the FSI. Enable role development and family care competencies.

Outcomes[3]

Implementation Outcomes

Fidelity (primary implementation outcome)
- Delivery
- Receipt
- Enactment

Feasibility
Acceptability
Appropriateness
Sustainability
Cost effectiveness

Contextual determinants
Implementation process and level of integration

Service Outcomes

Satisfaction with family care
Quality of communication
Support from nurses

Family member outcomes

Family functioning
Family resilience
Subjective well-being life satisfaction, quality of life
Psychological distress burden, depression, anxiety, postraumatic stress

FICUS Nurse-led Family Support Intervention

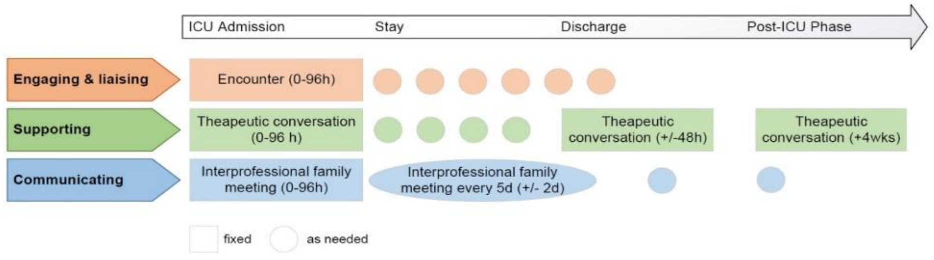

CFIR= Consolidated Framework for Implementation Reserach, FICUS= Family support intervention in intensive care units, FSI= Family support intervention, NPT= Normalization Process Theory

[1] Identified determinants (influencers) during context assessment before FICUS study start based on CFIR (0 neutral, + positive, - negativ, +/- positiv and negativ) (Damschroder et al., 2009), [2] Implementation strategies based on Naef et al. (2022), [3] Implementation strategy - mechanims based on NPT (May et al., 2009), [4] Measured outcomes according to the conceptualization of Proctor et al. (2009)

For peer review only - http://bmjopen.bmj.com/site/about/guidelines.xhtml

BMJ Open

Implementation of a multicomponent family support intervention in adult intensive care units – Study protocol for an embedded mixed methods multiple case study (FICUS Implementation Study)

Journal:	*BMJ Open*
Manuscript ID	bmjopen-2023-074142.R1
Article Type:	Protocol
Date Submitted by the Author:	26-Jul-2023

Complete List of Authors:	Oesch, Saskia; University of Zurich Faculty of Medicine, Institute for Implementation Science in Health Care; University Hospital Zurich, Center of Clinical Nursing Science<br>Verweij, Lotte; University of Zurich Faculty of Medicine, Institute for Implementation Science in Health Care; University Hospital Zurich, Center of Clinical Nursing Science<br>Clack, Lauren; University of Zurich Faculty of Medicine, Institute for Implementation Science in Health Care; University Hospital Zurich, Division of Infectious Diseases and Hospital Epidemiology<br>Finch, Tracy; Northumbria University, Nursing, Midwifery and Health<br>Riguzzi, Marco; University of Zurich Faculty of Medicine, Institute for Implementation Science in Health Care; University Hospital Zurich, Center of Clinical Nursing Science<br>Naef, Rahel; University of Zurich Faculty of Medicine, Institute for Implementation Science in Health Care; University Hospital Zurich, Center of Clinical Nursing Science

**Primary Subject Heading**:	Nursing
Secondary Subject Heading:	Intensive care, Nursing
Keywords:	Adult intensive & critical care < INTENSIVE & CRITICAL CARE, Protocols & guidelines < HEALTH SERVICES ADMINISTRATION & MANAGEMENT, Nursing Care

SCHOLARONE™
Manuscripts

Implementation of a multicomponent family support intervention in adult intensive care units – Study protocol for an embedded mixed methods multiple case study (FICUS Implementation Study)

Saskia Oesch RN MSc[1, 2], Lotte Verweij RN PhD[1,2], Lauren Clack PhD[1,3], Tracy Finch PhD[4], Marco Riguzzi PhD[1,2], Rahel Naef RN PhD[1,2]

[1]Institute for Implementation Science in Health Care, Faculty of Medicine, University of Zurich

[2]Center of Clinical Nursing Science, University Hospital Zurich

[3]Division of Infectious Diseases and Hospital Epidemiology, University of Zurich, University Hospital of Zurich, Zurich, Switzerland

[4]Department of Nursing, Midwifery & Health, Faculty of Health and Life Sciences, North Umbria University, Newcastle upon Tyne

Corresponding Author:

Saskia Oesch
University of Zürich
Institute for Implementation Science in Health Care
Faculty of Medicine
Universitätstrasse 84, 8006 Zürich
Switzerland
E-Mail: saskia.oesch2@uzh.ch
Phone: +41 79 673 90 87

For peer review only

For peer review only - http://bmjopen.bmj.com/site/about/guidelines.xhtml

Abstract

Background

The implementation of complex interventions is considered challenging, particularly in multi-site clinical trials and dynamic clinical settings. This study protocol is part of the FICUS hybrid effectiveness-implementation study. It aims to understand the integration of a multicomponent family support intervention in the real-world context of adult intensive care units. Specifically, the study will assess implementation processes and outcomes of the study intervention, including fidelity, and will enable explanation of the clinical effectiveness outcomes of the trial.

Methods and Analysis

This mixed methods multiple case study is guided by two implementation theories, the Normalization Process Theory and the Consolidated Framework for Implementation Research. Participants are key clinical partners and health care professionals of eight intensive care units allocated to the intervention group of the FICUS trial in the German-speaking part of Switzerland. Data will be collected at four time points over the 18-month active implementation and delivery phase using qualitative (small group interviews, observation, focus group interviews) and quantitative data collection methods (surveys, logs). Descriptive statistics and parametric and non-parametric tests will be used according to data distribution to analyze within and between cluster differences, similarities and factors associated with fidelity and the level of integration over time. Qualitative data will be analyzed using a pragmatic rapid analysis approach and content analysis.

Ethics and dissemination

Ethics approval was obtained from the Cantonal Ethics Committee of Zurich BASEC ID 2021-02300 (08. February 2022). Study findings will provide insights into implementation and its contribution to intervention outcomes, enabling understanding of the usefulness of applied implementation strategies and highlighting main barriers that need to be addressed for scaling the intervention to other health care contexts. Findings will be disseminated in peer-reviewed journals and conferences.

Protocol registration

Open science framework (OSF) https://osf.io/8t2ud Registered on 21 December 2022.

For peer review only

For peer review only - http://bmjopen.bmj.com/site/about/guidelines.xhtml

Strengths and limitations

- ➢ The use of hybrid study designs to understand intervention and implementation outcomes is key in complex intervention health research.
- ➢ The proposed study draws on established implementation science theories and methods to investigate the integration and delivery of a complex family support intervention in a highly dynamic clinical environment.
- ➢ A strength of this mixed-methods study is a balanced use of rigorous and pragmatic data collection and analysis strategies to inform ongoing implementation while also generating generalizable knowledge on implementation strategies and determinants around complex family support interventions.
- ➢ Given the anticipated small sample sizes per case, potential limitations will be a small or incomplete quantitative data set due to staff turnover or low response rate. Another potential limitation of the study might be the data collection burden on participants.
- ➢ The use of several data sources and the combination of qualitative and quantitative data will enable a comprehensive understanding of the phenomenon under study.

For peer review only

For peer review only - http://bmjopen.bmj.com/site/about/guidelines.xhtml

INTRODUCTION

Up to sixty percent of family members of critically ill patients in intensive care units (ICU) experience high levels of stress and uncertainty, which may lead to negative mental health outcomes (1-3). Families require consistent involvement, communication, and support, however, there is a lack of research on the clinical effectiveness and successful implementation of family-focused care in ICUs (3). To better support family health and to close this know-do gap in the area of family-focused ICU care, the FICUS study was launched to investigate the effectiveness and implementation of a nurse-led, interprofessional delivered family support intervention (FSI) in Swiss ICUs (4). Successful implementation of the three FSI components, namely family engagement, support, and communication by a family nurse and interprofessional team along the patient pathway is expected to increase the likelihood of clinical effectiveness, and will be critically influenced by the context into which the intervention is introduced, particularly as part of a multisite clinical trial (5-8).

Specific implementation guidance for integrating FSIs in critical health care settings is still scarce. Previously published research focusing on family nursing implementation have mainly focused on promoting nurses' skills development and open attitudes toward family-focused care (9-13). Main barriers to implement family-focused care in ICUs have been described as lack of understanding of what needs to be done to achieve family-centered care (e.g., lack of support from fellow nurses), organizational barriers (e.g., daily workload), individual barriers (e.g., health care professionals' attitudes), and barriers related to interprofessional care (e.g., communication barriers). In contrast, leadership support, clear and accurate communication, and engagement of the health care team are described as the most important facilitators (14-24). However, intervention research around FSIs in ICUs has not investigated their implementation (12, 25, 26), except for one ongoing trial, which will explore implementation processes and outcomes (27).

The implementation of complex interventions, such as FSIs, is challenging as they contain several independently and interdependently interacting components at the individual behavioral, interprofessional team, and organizational level (6-8, 28-30). Implementation of new interventions involves changes at the individual behavioral, team and organizational level (31) and several contextual determinants that influence successful integration may appear (32). A growing body of research focuses on the development of theories and frameworks to optimize the design and implementation of complex interventions (7, 8, 33, 34), but implementation success remains still widely variable (35). FSIs pose greater challenges for implementation evaluation and cannot rely on preestablished cause and effect specifications (36). As recommended within implementation science and complex intervention research, the focus should not only lie on the clinical effectiveness of a given intervention, but on the conditions needed to achieve the intervention's reach and impact in the real-world context (7, 8). Randomized controlled trials are important in establishing intervention effectiveness, however they focus solely on clinical health or service outcomes (37, 38). Even if

For peer review only

For peer review only - http://bmjopen.bmj.com/site/about/guidelines.xhtml

implementation outcomes, such as fidelity, serve as intermediate outcomes in studies of intervention effectiveness (39), in-depth exploration of implementation, particularly in the field of FSIs, has received scant attention (12, 40).

A focus on implementation is needed to learn about successful integration processes of FSIs in critical care settings. This paper reports an implementation study protocol for investigating the implementation process and outcomes of a multicomponent FSI alongside a cluster-randomized clinical trial (FICUS) (4), which examines the clinical effectiveness.

METHODS

Design

The current study is part of the FICUS hybrid effectiveness-implementation study and is conducted between June 2022 and January 2024. To investigate the implementation processes and outcomes of a complex phenomenon, that is the FSI in its "real-world" context (41), we will use an embedded multiple case study - mixed methods convergent design (42, 43), in which each of the participating ICUs is conceptualized as a "case" (44). Two research questions will be investigated:

1. What influence does the integration process of the FSI in eight ICUs have on the level of integration, mechanisms of impact and the implementation outcomes defined as fidelity, feasibility, acceptability, appropriateness, and sustainability?
2. Which contextual determinants (barriers and facilitators) influence the integration process and explain its impact on the intervention outcomes?

Patient and public involvement

Within the FICUS study, an advisory board was established with a patient expert, three family members, and a patient with life experience in critical care. The advisory board began its work with the research team during the application phase of the FICUS study and continues to actively participate in the study.

Study overview

Qualitative and quantitative data will be collected at four timepoints Figure 1. First, data will be collected as part of a formative process evaluation during the adaptive implementation process at three timepoints over 12 months (T1-T3). Rapid, primarily qualitative data collection procedures, such as small group interviews, observation and case conferences will be applied (45-47), coupled with structured self-assessment tools, and integrated into a series of meetings and activities with key clinical partners that serve to support implementation and to ensure quality of intervention delivery. A summative evaluation will be held with key clinical partners and ICU staff after the conclusion of the implementation / delivery phase (T4) to assess all study endpoints, using quantitative and qualitative assessment methods, such as focus group interviews and surveys.

Figure 1

review only

For peer review only - http://bmjopen.bmj.com/site/about/guidelines.xhtml

Guiding conceptual frameworks and theories

The Normalization Process Theory (NPT) (48, 49) and the Consolidated Framework for Implementation Research (CFIR) (50) will guide the evaluation of the implementation. While NPT explains changes in the way people think about and act to integrate a new intervention, CFIR categorizes and describes contextual determinants that influence implementation (51). NPT and CFIR will be combined and integrated in all stages of the research process, including conceptualization, data collection, analysis, and dissemination. Table 1 provides an overview and description of NPT and CFIR in the implementation study.

We developed an Implementation Research Logic Model (IRLM) drawing on CFIR (50), NPT (31), and Proctor's Conceptual Framework for Implementation Research (39) to develop a specific implementation theory around the FSI that outlines contextual determinants (52) implementation mechanisms of impact, and outcomes Figure 2. This approach aligns with the realist evaluation paradigm because it attends to context, mechanisms, and outcomes of a complex intervention implementation, but uses specific theories and frameworks from implementation science to do so (53-55).

Table 1 Selected implementation science frameworks and theories used within the FICUS implementation study

Framework / Theory	Type and description	Relevance to the FICUS Implementation Study
**NPT**	• The NPT is a middle-range sociological theory that conceptualizes implementation, embedding and integration of complex interventions in health care settings (49).<br>• It focuses on the work required to embed and normalize an intervention in routine practice. NPT consists of 4 key constructs (coherence, cognitive participation, collective action & reflexive monitoring) and 16 sub-constructs (56).	• NPT provides a flexible framework to evaluate integration and embedding of the FSI.<br>• NPT will help to first, better understand the way implementation processes and context shape each other, and second, to explore the collaborative work people do to make sense of the FSI and to integrate it in ICU clinicians' daily routine (57).<br>• NPT will be used to investigate and to describe the integration and normalization of the FSI in ICU teams.

For peer review only

For peer review only - http://bmjopen.bmj.com/site/about/guidelines.xhtml

Framework / Theory	Type and description	Relevance to the FICUS Implementation Study
**CFIR[1]**	• The CFIR is a meta-theoretical determinant framework which provides a pragmatic structure to guide process evaluations.<br>• CFIR provides an overarching typology to promote implementation theory development and verification about what works where and why across multiple contexts (50).<br>• It includes five domains (inner setting, outer setting, intervention characteristics, characteristics of individuals involved and processes of implementation) (50).<br>• Within the five domains are 37 constructs that can each act as a barrier and / or facilitator to the implementation of an intervention.	• The CFIR framework includes a wide range of constructs that relate not only to individual characteristics, but also to structural characteristics of the health care system, as well as on characteristics of the intervention itself.<br>• CFIR provides a degree of flexibility where constructs can be selected that are the most relevant to understand individual and system barriers and facilitators associated with the implementation (50) and to assess key contextual determinants to consider within the outer setting and the intervention characteristics domains, which are outside of the NPT scope (51).<br>• CFIR will be used to describe potential influencing determinants (barriers and facilitators) during implementation.

[1] We will use the first version of CFIR (50) because our contextual analysis to adjust the implementation (52) was conducted before the publication of CFIR version 2.0 (58).

Figure 2

Setting

The FSI will be implemented in eight of sixteen ICUs participating in the FICUS trial, which have been randomly assigned to the intervention arm (4). All ICUs are certified by the Swiss Society for Intensive Medicine; two are situated in a university-affiliated, and six in a cantonal or teaching hospital.

Participants and sampling

Data will be collected from key clinical partners (KP) and ICU health care professionals (HP) by a senior researcher and a doctoral student. The senior researcher is trained and skilled in qualitative data collection and will train and supervise the doctoral student in doing so. KP are local clinical and management staff involved in the implementation and delivery of the FSI (n=45) and will include local implementers (LI) who support the implementation process, trained family nurses (FN), who are registered nurses with a certification in ICU nursing or equivalent, who are trained in family systems nursing and deliver the FSI, as well as a nurse and physician team leaders and/or their deputy (TL). Quantitative and qualitative data will be gathered from all KP during the process evaluation. The second participant group will include HP working on the participating ICUs (approximate n ≈ 750). Quantitative data for the summative evaluation will be gathered from all HP with an anticipated response rate of 50-70% using a comprehensive sampling strategy. To increase response rates, 3-weekly reminder e-mails will be sent twice. Qualitative data will be gathered from a subsample of HP (n= 80-100) using a purposive sampling strategy. Inclusion criteria will be: ICU physicians or nurses working clinically on study units, having an active or influential role in the

For peer review only - http://bmjopen.bmj.com/site/about/guidelines.xhtml

implementation / delivery of the FSI and / or having either knowledge / experience with the FSI and / or a clinical leadership responsibility, having worked at the ICU for at least 6 months.

Study endpoints

The study endpoints pertain to three conceptual dimensions: 1) process indicators, 2) implementation outcomes and 3) contextual determinants. Table 2 provides a summary of the study endpoints, operationalization, and data collection timepoints. A more detailed description of the study endpoints, definitions, data source, operationalization and data collection timepoints is available as supplementary material.

Process indicators

The implementation process and integration mechanisms will be investigated to gain an in-depth understanding of the implementation from the perspective of KP. It is of particular interest how the FSI becomes (or fails to become) integrated into interprofessional care delivery to families and a normalized ICU care process (48). Indicators will include self-perceived integration of the intervention in the interprofessional ICU team and the overall level of integration in ICU processes.

Implementation outcomes

We will investigate the implementation outcomes of fidelity, feasibility, acceptability, appropriateness and sustainability (39). As fidelity is vital to evaluate our intervention and implementation theory for predicting effectiveness outcomes (59, 60), we chose it as the primary outcome. Fidelity is defined according to the Conceptual Framework for Implementation Fidelity (61, 62), and has been operationalized within the three domains of delivery, receipt, and enactment as outlines by Bellg and colleagues (63).

We will investigate feasibility, appropriateness, and acceptability because these outcomes play a critical role in implementation research and serve as indicators of the effect of the implementation process and as prerequisites for achieving the desired service and family outcomes (64). To understand how the implementation develops over time and how to best support long-term delivery, we will investigate sustainability as well as post- implementation sustainment outcomes (65).

Contextual determinants

Contextual determinants at the level of individuals, the team, the organization, and the implementation itself, namely barriers and facilitators that hinder or support successful implementation, will be assessed from a clinician's perspective guided by the CFIR (50) and NPT (48)

Table 2 Summary study endpoints, operationalization and data collection timepoints

STUDY ENDPOINTS	OPERATIONALIZATION	DATA COLLECTION TIMEPOINT			
		T1	T2	T3	T4
<u>**Implementation Process**</u>					

For peer review only - http://bmjopen.bmj.com/site/about/guidelines.xhtml

STUDY ENDPOINTS	OPERATIONALIZATION	DATA COLLECTION TIMEPOINT			
		T1	T2	T3	T4
Process indicators	Small group interviews[1]	x		x	
	NoMAD [1, 2, 3]			x	x
	Implementation rating tool [1]	x		x	
**Implementation Outcomes**					
**Fidelity: Delivery**					
Relative frequency	Intervention fidelity log[1,9]	x	x	x	x
Absolute dose	Intervention fidelity log[1,9]	x	x	x	x
Consistency	Intervention fidelity log[1,9]	x	x	x	x
Availability	Intervention capacity assessment[1]	x			
	Focus group interviews[2]				x
**Fidelity: Receipt**					
Attendance at case conferences	Case conferences[1,4]	x	x	x	x
Comprehension & skills	FNPS[1, 5]	x	x		x
	Focus group interviews[2]				x
**Fidelity Enactment**					
Engagement & performance	Fidelity Self-Rating Tool[1]		x		x
	Semi-structured observation[1]		x		
	Focus group interviews[2]				x
**Feasibility**	FIM[1,2,6]	x		x	x
	Focus group interviews[2]				x
**Acceptability**	AIM[1,2,7]	x		x	x
	Focus group interviews				x
**Appropriateness**	IAM[1,2,8]	x		x	x
	Focus group interviews[2]				x
**Sustainability / Sustainment**	Sustainability implementation survey[10]				x
	Focus group interviews[2]				x
**Contextual Determinants**	Small group interviews[1]	x		x	
	Focus group interviews[2]				x

Abbreviations: FN Family Nurses, LI local implementers, KP Key clinical partners, HP Health Care Professionals, ICU Intensive Care Unit, LoS Length of stay, CFIR Consolidated Framework for Implementation Research, NPT Normalization Process Theory

[1] Part of the process evaluation,[2] Part of the summative evaluation

[3] German Version of the Normalization Measure Development Questionnaire (NoMAD) (66)

[4] Monthly case conferences throughout the 18-months active implementation and delivery phase

[5] German version of the Family Nursing Practice Scale (FNPS) (67)

[6] German version of the Feasibility Intervention Measure (FIM) (68)

[7] German version of the Acceptability Intervention Measure (AIM) (68)

[8] German version of the Intervention Appropriateness Measure (68)

[9] Case report forms embedded in RED Cap (69) and completed throughout the study by family nurses / interventionists

[10] Data collection occurs 6 and 12 months after active implementation and intervention delivery / FICUS study end

Qualitative data collection

Small group interview

Small group interviews refer to a semi-structured discussion with a small group of KP (4 to 5 participants) to facilitate effective discussion on the implementation process and ensure that each KP is adequately heard. This method will allow for gathering information, exploring opinions, and understanding perspectives (70). A total of 16 small group interviews will be held with KP as part of the process evaluation to assess contextual determinants (barriers and facilitators) and processes that influence the implementation. An interview guide, which is based on NPT and CFIR will be used to explore participants' understanding and perceptions of the intervention delivery, their team progress to achieve integration, and the barriers and facilitators they currently face (57). Small group

For peer review only

For peer review only - http://bmjopen.bmj.com/site/about/guidelines.xhtml

interviews will include questions such as: Was the defined implementation strategy useful, and do any adjustments or additional measures need to be taken? What are the reasons that currently hinder the implementation of the FSI, and what have been the biggest challenges so far and what are some ideas on how to overcome these barriers in the next phase of implementation? and, is the FSI capable of addressing the needs of the families and patients and how well does the FSI fit into current practices and processes, and does it require any adjustments? All interviews will be held face-to-face by two researchers at the respective ICU, audio-recorded, and expected to last 60-90 minutes. Immediately after each interview, key topics will be summarized in a protocol and provided to the KP for validation. This form of member checking will give them the ability to correct errors and wrong interpretations and to create trustworthiness (43) .

Focus group interview

Focus group interviews differ from small group interviews as they are more structured and conducted with a larger number of participants (8-10 participants) to obtain a comprehensive picture of the FSI implementation (70). A total of ten focus group interviews will be held as part of the summative evaluation with KP and HP as it is a useful way of collecting qualitative data as social interactions, shared values, cultures and practices which are of particular interest (71).

First, one focus group interview will be held on each ICU with a purposive sample of 8-10 KS and HP, which represent different professional groups, roles, and levels of involvement in the intervention and implementation. A semi-structured interview guide will be used including open-ended questions focusing on: How do ICU staff describe the implementation and feasibility of the FSI on their ICU, what team processes and implementation activities took place during the implementation of the FSI? What factors were supportive or inhibiting and how did the team perceive the role of FN's and the family care pathway? and what impact (benefits, unexpected consequences) did they experience in their daily work, interprofessional and with patients and their families?

In addition, two focus group interviews will be held with 8-10 family nurses using an interview guide that includes open-ended questions on FN's experiences and perspectives on their role adoption, team-based intervention delivery and clinical work with families, experiences with FSI implementation in the study context, observed benefits for themselves, the team, and the families, as well factors that promoted or hindered intervention delivery and integration into ICU care.

All focus group interviews will be held face-to-face by two researchers and will take place on site at the respective ICU or an accessible location, in a quiet room to avoid interruptions. Interviews will be digitally recorded, transcribed, and field notes will be taken. The interviews and the field notes will be transcribed, checked for accuracy, anonymized, and entered into MAXQDA for analysis.

Observation of intervention delivery

A total of eight semi-structured observations of intervention delivery by trained FN, that is, one per ICU, will be conducted to assess intervention fidelity and the establishment of structures for the FSI

For peer review only - http://bmjopen.bmj.com/site/about/guidelines.xhtml

using a semi-structured fidelity audit observation tool. The tool bases on the intervention manual and was developed by the research team. It consists of four items on available structures for the FICUS study and 59 FSI activities corresponding to the three main components 1) engaging and liaising, 2) supporting and 3) communicating. Activities will be rated according to their performance by the FN (performed, partially performed, not performed, not applicable). In addition, the timepoint of the conversation (e.g., admission) and the type of conversation (e.g., on-site, via telephone) will be documented. An additional text box at the end of the tool will allow to document observations about the ICU context. The tool was pretested once by one researcher. All observations will be conducted by a doctoral student or a researcher, which are both trained in family systems nursing and are expected to last 2-4 hours each.

Case conference

Monthly case conferences with FNs will be held as moderated group discussions to ensure quality of FSI delivery and to support consistency and adherence to the FSI activities. During case conferences, a specific family case will be presented by FN's and FN's will have the opportunity to discuss, to share their experiences and to reflect on the presented case. Based on the discussions, field notes will be taken by a doctoral student and FNs' participation rate will be captured, as it is an indicator for fidelity receipt. Participation of the FN will be documented in a structured participation form. All case conferences will be held online by two senior researchers trained in family systems nursing and will last 60 minutes.

Quantitative measures

Fidelity Self-Rating Tool (Fidelity-SRT)

To capture quality and consistency of intervention performance (fidelity enactment), FN's appraisal of self-perceived engagement and performance in FSI delivery will be assessed two times using the Fidelity-SRT. The tool is based on the intervention manual and was developed by the FICUS research team. It includes 3 subscales according to the intervention components: 1) engaging and liaising (15 items), 2) supporting (27 items) and 3) communicating (15 items). All items will be rated on 5- point Likert scale (1 = never to 5 = always) regarding the last 3-5 families taken care of by the FN. A low mean score indicates low self-perceived success in FSI performance whereas a higher score means higher succeed in intervention delivery. The Fidelity-SRT was pretested by two KP and requires approximately 10 minutes to complete.

Intervention Capacity Assessment (ICA)

To assess FN's availability as defined per trial protocol to ensure provision of intervention capacity (fidelity delivery), the ICA will be completed two times during process evaluation by KP's. The ICA consists of two self-developed items referring to the four weeks prior to the assessment and include: 1) Was there always at least one FN available to deliver the FSI during five days per week? 2) (only for

For peer review only - http://bmjopen.bmj.com/site/about/guidelines.xhtml

FN) To what extent was it possible to provide the FSI to family members according to the timeline requirements of the protocol and the needs of the family? Both items will be rated on a 5-point Likert scale ranging from 1 (never) to 5 (always).

Intervention Fidelity Logs (IFL)

FSI characteristics, such as duration in minutes, frequency, delivery mode, intervention activity (as defined in the intervention manual) will be recorded in a structured, online intervention log, which is part of the clinical trial electronic data capture system REDCap (69). The logs are completed by FN for each included trial participant on an ongoing basis.

Family Nursing Practice Scale (FNPS)

To assess FN's appraisal of practice skills and their reflection in working with families, the 10-item German version of the "Family Nursing Practice Scale" will be used (67). Items will be scored on a 5-point Likert-type scale ranging from one (high level) to five (low level). Lower mean scores will represent higher practice skills. The FNPS was validated in critical care, suggesting high internal consistency (Crohnbach's alpha 0.84) (67)

Feasibility Intervention Measure (FIM), Acceptability of Intervention Measure (AIM) and Intervention Appropriateness Measure (IAM)

To assess KPs' perceived level of feasibility, acceptability, and appropriateness of the FSI, adapted psychometrically validated German versions of the FIM, AIM and IAM will be completed three times. Each of these surveys include four items that utilize a 5-point Likert scale ranging from one (completely disagree) to five (completely agree). A recurring adaption is made by inserting "FSI" into the appropriate space on the instrument e.g., "(insert Intervention) is doable" will become "FSI is doable". All three surveys are reliable and pragmatic instruments with a high Crohnbach's alpha (FIM α = .89; AIM α = .80, IAM; α= 0.87) (64, 68).

Normalization Measure Development questionnaire (NoMAD)

To assess HP's perceived level of integration, the German version of the NoMAD will be used two times (66). It includes 20 items utilizing a 5-point Likert scale ranging from one (completely disagree) to five (completely agree) and is adaptable by specifying the "(the intervention)" to study purpose (72), that is, the "FSI". The NoMAD instrument has high face validity, construct validity and internal consistency for assessing perceptions of factors relevant to the integration of the FSI. Internal consistency (Crohnachs's alpha) was reported as follows: coherence (4 items, α=0.71); collective action (7 items, α= 0.78); cognitive participation (4 items, α= 0.81) and reflexive monitoring (5 items, α= 0.65). Overall, the normalization scale was described as highly reliable (20 items, α= 0.89) (72).

Sustainability Implementation Survey

To assess both, KP's appraisal of sustainability determinants and post- implementation sustainment outcomes, a self-developed sustainability survey will be sent at six and twelve months after the last

For peer review only - http://bmjopen.bmj.com/site/about/guidelines.xhtml

intervention delivery and before the follow-up completion by the last study participant. The survey will include questions about the extent to which the FSI is still delivered as intended after FICUS study support is terminated (sustainability) and continued enactment of processes and practices in daily ICU routine that have been conveyed and learned through the FSI (73). All items will be rated on a 5-point Likert scale ranging from one (completely disagree) to five (completely agree).

Demographics

Information on participants' age, gender, profession, institution, level of employment, level of education, work experience in the ICU, overall work experience in the profession and whether family nursing was part of the curriculum of their training and/or studies, will be collected. Furthermore, cluster data at ICU level will be collected to describe the eight cases in detail. Cluster data will include ICU cluster size (number of health care professionals), number of beds, number of admissions per year, and average length of stay.

All surveys will be implemented in UNIPARK software.

Data analysis

Qualitative data analysis

Data analysis of the process evaluation will require prompt analysis of interview data to rapidly develop or modify implementation strategies in case of need. Hence, small group interviews will be analyzed using an adapted approach of Rapid Identification of Themes from Audio recordings (RITA) (74), in combination with a deductive approach guided by CFIR / NPT (47). This form of directed content analysis will apply the following steps: 1) specifying the evaluation foci, 2) identifying themes from audio recordings, 3) creating a codebook, 4) coding and refining the codebook and 5) sorting codes into themes inductively and deductively drawing on CFIR and NPT constructs (75).

Qualitative data from the summative evaluation (T4) will be analyzed using content analysis, which allows the use of deductive and inductive strategies. It uses the following steps: 1) reading each of the transcribed interviews to get familiar with the data, taking notes of first impressions to gain a sense of the whole phenomenon, 2) identifying and developing codes for each unit of meaning, 3) developing of definitions for each code and group them into categories of related meanings, and 4) synthesizing codes and categories into themes (75, 76).

Quantitative data analysis

Raw data will be verified, encrypted, and entered into R Project for analysis. Descriptive statistics will be computed for the entire sample, by case and by data collection timepoint. Depending on scale level and distribution, results will be presented either as absolute and relative frequencies, as mean and standard deviation, or as median and interquartile range. If scale levels and distributions allow, differences in central tendency will be tested by Student's t-test (paired or unpaired), with Welch's correction in the case of variance heterogeneity (unpaired case). Otherwise, differences in the

For peer review only - http://bmjopen.bmj.com/site/about/guidelines.xhtml

distribution of ordinal data will be tested by Wilcoxon's signed-rank test (paired case within the same clusters or individuals over two timepoints, e.g., Fidelity-SRT or NoMAD; unpaired case be-tween clusters or individuals, e.g., FNPS at baseline, referred to as Mann & Whitney's U-test). For nominal comparisons between clusters or individuals (unpaired), a chi-squared test will be used if observed and expected frequencies allow for it, and Fisher's exact test will be used otherwise. For nominal comparisons within clusters or individuals over different timepoints (paired), McNemar's test will be used (or Cochran's Q test if a binary attribute is compared over more than two matched observations) (77).

Mixed-methods and cross-case analysis

To provide an in-depth understanding of the phenomenon under study, and to increase the validity of our findings, data triangulation will occur on case level by comparing qualitative and quantitative data sets (44, 78). Data comparison occurs after the separate analysis of qualitative and quantitative data (43, 79, 80). Datasets will be merged by listing and joining results on a same table, summarizing raw data that are important to consider and displaying statistics and themes of each case in a matrix. Qualitative data will be mixed with quantitative data in relation to the implementation outcomes (i.e., qualitative data on acceptability with IAM data) and the implementation process (i.e., qualitative data on integration with NoMAD data) using comparing, contrasting, and constantly verifying methods (80).

Integration will also occur through construction of cases to develop a robust case presentation (42, 78). We will compare-contrast cases to not only identify patterns within but also across cases to build explanations for a successful implementation. To compare different cases, cross-case synthesis will be used, to compare and to identify similarities and differences between the cases. This will involve tables that display the data according to a uniform framework to compare across cases for both research questions and site-specific experiences to emerge from the data (44). Finally, data will be brought together in a logic model including moderating influencers (81).

Data management

Intervention log data is stored on a MySQL database server hosted by the Clinical Trials Unit Zurich, which holds a REDCap End-User License Agreement for this electronic data capture system (4). Unencrypted primary data, such as fieldnotes and audiotapes, will be stored in a lockable compartment to which only the research team has access. All documents are kept encrypted on the University of Zurich server and the key will be destroyed after completion of the study. Essential documents will be retained for at least 10 years after termination of the study.

CONTRIBUTION TO THE LITERATURE AND DISSEMINATION

This protocol outlines the rationale, design and methods for a process and outcome evaluation of a new, multicomponent, nurse-led FSI in ICUs. The key feature of the study design is the development of flexible and pragmatic data collection methods to capture data across five areas: 1) if the FSI was

For peer review only

For peer review only - http://bmjopen.bmj.com/site/about/guidelines.xhtml

delivered as intended (fidelity), 2) how clinicians experienced the implementation process of the FSI , 3) which determinants (barriers and facilitators) influenced the integration process, 4) the impact of tailored implementation strategies to overcome contextual determinants and 5) to explain / interpret the clinical effectiveness results. Findings will be used to build a comprehensive understanding of how and why the FSI in ICUs was effectively implemented and delivered, and to delineate recommendations for scale-up to further ICUs and / or adaptions to different clinical contexts. Study findings will make a significant contribution to the current body of knowledge in attending to implementation of FSIs, as there is only currently one study protocol by Curtis et al. (27) outlining plans to explore implementation. This study protocol may serve as a useful guidance for planning similar studies and projects that aim to test and / or implement complex interventions in real-world contexts, such as a nurse-led family support program in ICUs.

Our findings will be disseminated to all partners and stakeholders involved and interested in its long-term implementation. Research findings will be disseminated via peer-reviewed journals and conferences.

ETHICS

The FICUS study, including the embedded implementation study has been approved by the responsible Swiss cantonal ethics committees (Nr. 2021-02300). Study participants will be informed about the purpose of this study and study participation orally and received a study information pack. Relevant national and international data protection regulation will be respected in accordance with the principles of the Declaration of Helsinki (82).

Abbreviations

AIM: Acceptability Intervention Measure
CC: Case Conferences
CFIR: Consolidated Framework for Implementation Research
FICUS: Family Intensive Care Units
Fidelity-SRT: Fidelity Self Rating Tool
FIM: Feasibility Intervention Measure
FN: Family Nurses
FNPS: Family Nursing Practice Scale
FSI: Family Support Intervention
HP: Health Care Professionals
IAM: Intervention Appropriateness Measure
ICA: Intervention Capacity Assessment
ICU: Intensive Care Unit
IFL: Intervention Fidelity Logs
KP: Key Clinical Partners
LI: Local Implementers
NoMAD: Normalization Measure Development Questionnaire
NPT: Normalization Process Theory
OSF: Open Science Framework
PICS-F: Post-Intensive Care Syndrome-Family
RITA: Rapid Identification of Themes from Audio recordings
TL: Team Leaders

For peer review only - http://bmjopen.bmj.com/site/about/guidelines.xhtml

Consent for publication

Not applicable.

Availability of data and materials

Data sharing is not applicable to this article as no datasets were generated or analyzed during the current study (study protocol).

Competing interests

The authors declare that they have no competing interests.

Funding

The study is funded by the Swiss National Science Foundation (SNSF, No. 33IC30_198778/1). The funder has no role in the development of the study design; collection, analysis, or interpretation of the data; writing of the manuscript; or decision to submit the manuscript for publication.

Authors' contributions

SO, LV and RN conceptualized the study. The manuscript was written by SO and LV. RN provided ongoing feedback and critically revised the manuscript. MR supported the designing of the statistical analysis plan. TF and LC critically reviewed the study design and manuscript for important intellectual content. All authors have reviewed the drafts and approved the final version.

Acknowledgements

We are very grateful to the FICUS study group for supporting this study.

References

1. Davidson JE, Aslakson RA, Long AC, Puntillo KA, Kross EK, Hart J, et al. Guidelines for Family-Centered Care in the Neonatal, Pediatric, and Adult ICU. Critical care medicine. 2017;45(1):103-28.
2. Gerritsen RT, Hartog CS, Curtis JR. New developments in the provision of family-centered care in the intensive care unit. Intensive Care Medicine. 2017;43(4):550-3.
3. Kiwanuka F, Sak-Dankosky N, Alemayehu YH, Nanyonga RC, Kvist T. The evidence base of nurse-led family interventions for improving family outcomes in adult critical care settings: A mixed method systematic review. International Journal of Nursing Studies. 2022;125:104100.
4. Naef R, Filipovic M, Jeitziner M-M, von Felten S, Safford J, Riguzzi M, et al. A multicomponent family support intervention in intensive care units: study protocol for a multicenter cluster-randomized trial (FICUS Trial). Trials. 2022;23(1):533.
5. May C, Finch T, Mair F, Ballini L, Dowrick C, Eccles M, et al. Understanding the implementation of complex interventions in health care: the normalization process model. BMC Health Services Research. 2007;7(1):148.
6. Moore GF, Audrey S, Barker M, Bond L, Bonell C, Hardeman W, et al. Process evaluation of complex interventions: Medical Research Council guidance. BMJ : British Medical Journal. 2015;350:h1258.
7. Pfadenhauer LM, Gerhardus A, Mozygemba K, Lysdahl KB, Booth A, Hofmann B, et al. Making sense of complexity in context and implementation: the Context and Implementation of Complex Interventions (CICI) framework. Implementation Science. 2017;12(1):21.
8. Skivington K, Matthews L, Simpson SA, Craig P, Baird J, Blazeby JM, et al. A new framework for developing and evaluating complex interventions: update of Medical Research Council guidance. Bmj. 2021;374:n2061.

For peer review only - http://bmjopen.bmj.com/site/about/guidelines.xhtml

9. Duhamel F, Dupuis F, Turcotte A, Martinez AM, Goudreau J. Integrating the Illness Beliefs Model in clinical practice: a Family Systems Nursing knowledge utilization model. Journal of Family Nursing. 2015;21(2):322-48.

10. Goldfarb M, Bibas L, Burns K. Patient and Family Engagement in Care in the Cardiac Intensive Care Unit. Canadian Journal of Cardiology. 2020;36(7):1032-40.

11. Naef R, Kaeppeli BM, Lanter R, Petry H. Implementing Family Systems Care Through an Educational Intervention With Nurses and Midwives in Obstetrics and Gynecological Care: A Mixed-Methods Evaluation. Journal of Family Nursing. 2020;26(2):138-52.

12. White DB, Angus DC, Shields AM, Buddadhumaruk P, Pidro C, Paner C, et al. A Randomized Trial of a Family-Support Intervention in Intensive Care Units. The New England Journal of Medicine. 2018;378(25):2365-75.

13. Svavarsdottir EK, Sigurdardottir AO, Konradsdottir E, Stefansdottir A, Sveinbjarnardottir EK, Ketilsdottir A, et al. The process of translating family nursing knowledge into clinical practice. Journal of Nursing Scholarship. 2015;47(1):5-15.

14. Kiwanuka F, Shayan SJ, Tolulope AA. Barriers to patient and family-centred care in adult intensive care units: A systematic review. Nurs Open. 2019;6(3):676-84.

15. Hamilton R, Kleinpell R, Lipman J, Davidson JE. International facilitators and barriers to family engagement in the ICU: Results of a qualitative analysis. Journal of Critical Care. 2020;58:72-7.

16. Price AM, McAndrew NS, Thaqi Q, Kirk M, Brysiewicz P, Eggenberger S, et al. Factors influencing critical care nurses' family engagement practices: An international perspective. Nursing in Critical Care. 2022;n/a(n/a).

17. Naef R, Brysiewicz P, Mc Andrew NS, Beierwaltes P, Chiang V, Clisbee D, et al. Intensive care nurse-family engagement from a global perspective: A qualitative multi-site exploration. Intensive and Critical Care Nursing. 2021;66:103081.

18. Imanipour M, Kiwanuka F. Family nursing practice and family importance in care – Attitudes of nurses working in intensive care units. International Journal of Africa Nursing Sciences. 2020;13:100265.

19. Esmaeili M, Cheraghi MA, Salsali M. Explaining the Context of Patient-Centered Care in Critical Care Units. Journal of Holistic Nursing and Midwifery. 2017;27(3):17-26.

20. Thirsk LM, Vandall-Walker V, Rasiah J, Keyko K. A Taxonomy of Supports and Barriers to Family-Centered Adult Critical Care: A Qualitative Descriptive Study. Journal of Family Nursing. 2021;27(3):199-211.

21. Hetland B, Hickman R, McAndrew N, Daly B. Factors Influencing Active Family Engagement in Care Among Critical Care Nurses. AACN Advanced Critical Care. 2017;28(2):160-70.

22. Kleinpell R, Zimmerman J, Vermoch KL, Harmon LA, Vondracek H, Hamilton R, et al. Promoting Family Engagement in the ICU: Experience From a National Collaborative of 63 ICUs. Critical Care Medicine. 2019;47(12):1692-8.

23. Burns KEA, McDonald E, Debigaré S, Zamir N, Vasquez M, Piche-Ayotte M, et al. Patient and family engagement in patient care and research in Canadian intensive care units: a national survey. Canadian Journal of Anesthesia/Journal canadien d'anesthésie. 2022;69(12):1527-36.

24. Thürlimann E, Verweij L, Naef R. The Implementation of Evidence-Informed Family Nursing Practices: A Scoping Review of Strategies, Contextual Determinants, and Outcomes. Journal of Family Nursing. 2022:10748407221099655.

25. Curtis R, Treece PD, Nielsen EL, Gold J, Ciechanowski PS, Shannon SE, et al. Randomized Trial of Communication Facilitators to Reduce Family Distress and Intensity of End-of-Life Care. American Journal of Respiratory and Critical Care Medicine. 2016;193(2):154-62.

26. Kentish-Barnes N, Chevret S, Valade S, Jaber S, Kerhuel L, Guisset O, et al. A three-step support strategy for relatives of patients dying in the intensive care unit: a cluster randomised trial. Lancet. 2022;399(10325):656-64.

27. Curtis JR, Kentish-Barnes N, Brumback LC, Nielsen EL, Pollak KI, Treece PD, et al. Facilitating communication for critically ill patients and their family members: Study protocol for two randomized trials implemented in the U.S. and France. Contemporary clinical trials. 2021;107:106465.

For peer review only - http://bmjopen.bmj.com/site/about/guidelines.xhtml

28. Campbell M, Fitzpatrick R, Haines A, Kinmonth AL, Sandercock P, Spiegelhalter D, et al. Framework for design and evaluation of complex interventions to improve health. BMJ. 2000;321(7262):694-6.

29. Nilsen P, Bernhardsson S. Context matters in implementation science: a scoping review of determinant frameworks that describe contextual determinants for implementation outcomes. BMC Health Services Research. 2019;19(1):189.

30. May CR, Johnson M, Finch T. Implementation, context and complexity. Implementation Science. 2016;11(1):141.

31. May CR, Mair F, Finch T, MacFarlane A, Dowrick C, Treweek S, et al. Development of a theory of implementation and integration: Normalization Process Theory. Implementation Science. 2009;4(1):29.

32. Groot Kormelinck CM, Janus SIM, Smalbrugge M, Gerritsen DL, Zuidema SU. Systematic review on barriers and facilitators of complex interventions for residents with dementia in long-term care. International Psychogeriatrics. 2021;33(9):873-89.

33. Michie S, Johnston M, Abraham C, Lawton R, Parker D, Walker A. Making psychological theory useful for implementing evidence based practice: a consensus approach. Quality & Safety In Health Care. 2005;14(1):26-33.

34. Nilsen P. Making sense of implementation theories, models and frameworks. Implementation Science. 2015;10(1):53.

35. Presseau J, Grimshaw JM, Tetroe JM, Eccles MP, Francis JJ, Godin G, et al. A theory-based process evaluation alongside a randomised controlled trial of printed educational messages to increase primary care physicians' prescription of thiazide diuretics for hypertension [ISRCTN72772651]. Implementation Science. 2016;11(1):121.

36. Kainz K, Metz A, Yazejian N. Tools for Evaluating the Implementation of Complex Education Interventions. American Journal of Evaluation. 2021;42(3):399-414.

37. Moore GF, Evans RE. What theory, for whom and in which context? Reflections on the application of theory in the development and evaluation of complex population health interventions. SSM - Population Health. 2017;3:132-5.

38. Saunders RP, Evans MH, Joshi P. Developing a process-evaluation plan for assessing health promotion program implementation: a how-to guide. Health Promotion Practice. 2005;6(2):134-47.

39. Proctor E, Silmere H, Raghavan R, Hovmand P, Aarons G, Bunger A, et al. Outcomes for implementation research: conceptual distinctions, measurement challenges, and research agenda. Administration and Policy in Mental Health. 2011;38(2):65-76.

40. Kentish-Barnes N, Chaize M, Seegers V, Legriel S, Cariou A, Jaber S, et al. Complicated grief after death of a relative in the intensive care unit. European Respiratory Journal. 2015;45(5):1341-52.

41. Paparini S, Green J, Papoutsi C, Murdoch J, Petticrew M, Greenhalgh T, et al. Case study research for better evaluations of complex interventions: rationale and challenges. BMC Medicine. 2020;18(1):301.

42. Guetterman TC, Fetters MD. Two Methodological Approaches to the Integration of Mixed Methods and Case Study Designs: A Systematic Review. American Behavioral Scientist. 2018;62(7):900-18.

43. Creswell JW. A Concise Introduction to Mixed Methods Research. 2 ed: SAGE Publications, Inc; 2021.

44. Yin RK. Case Study Research and Applications

COSMOS Corporation; 2018. 319 p.

45. Vindrola-Padros C, Vindrola-Padros B. Quick and dirty? A systematic review of the use of rapid ethnographies in healthcare organisation and delivery. BMJ Quality & Safety Journal. 2018;27(4):321-30.

46. Palinkas LA, Zatzick D. Rapid Assessment Procedure Informed Clinical Ethnography (RAPICE) in Pragmatic Clinical Trials of Mental Health Services Implementation: Methods and Applied Case Study. Administration and policy in mental health. 2019;46(2):255-70.

47. Nevedal AL, Reardon CM, Opra Widerquist MA, Jackson GL, Cutrona SL, White BS, et al. Rapid versus traditional qualitative analysis using the Consolidated Framework for Implementation Research (CFIR). Implementation Science. 2021;16(1):67.

For peer review only - http://bmjopen.bmj.com/site/about/guidelines.xhtml

48. May CR, Cummings A, Girling M, Bracher M, Mair FS, May CM, et al. Using Normalization Process Theory in feasibility studies and process evaluations of complex healthcare interventions: a systematic review. Implementation Science. 2018;13(1):80.

49. May C, Finch T. Implementing, Embedding, and Integrating Practices: An Outline of Normalization Process Theory. Sociology. 2009;43(3):535-54.

50. Damschroder LJ, Aron DC, Keith RE, Kirsh SR, Alexander JA, Lowery JC. Fostering implementation of health services research findings into practice: a consolidated framework for advancing implementation science. Implementation Science. 2009;4(1):50.

51. Schroeder D, Luig T, Finch TL, Beesoon S, Campbell-Scherer DL. Understanding implementation context and social processes through integrating Normalization Process Theory (NPT) and the Consolidated Framework for Implementation Research (CFIR). Implementation Science Communications. 2022;3(1):13.

52. Verweij L, Oesch S, Naef R. Tailored Implementation of the FICUS multicomponent family support intervention in adult intensive care units: Findings from a mixed methods contextual analysis [Manuscript submitted for publication]. Implementation Science 2023.

53. Dalkin SM, Hardwick RJL, Haighton CA, Finch TL. Combining Realist approaches and Normalization Process Theory to understand implementation: a systematic review. Implementation Science Communications. 2021;2(1):68.

54. Pawson R, Tilley N. An introduction to scientific realist evaluation. Evaluation for the 21st century: A handbook. Thousand Oaks, CA, US: Sage Publications, Inc; 1997. p. 405-18.

55. Palm R, Hochmuth A. What works, for whom and under what circumstances? Using realist methodology to evaluate complex interventions in nursing: A scoping review. International Journal of Nursing Studies. 2020;109:103601.

56. Finch TL, Mair FS, O'Donnell C, Murray E, May CR. From theory to 'measurement' in complex interventions: methodological lessons from the development of an e-health normalisation instrument. BMC medical research methodology. 2012;12:69-.

57. May CR, Albers B, Bracher M, Finch TL, Gilbert A, Girling M, et al. Translational framework for implementation evaluation and research: a normalisation process theory coding manual for qualitative research and instrument development. Implementation Science. 2022;17(1):19.

58. Damschroder LJ, Reardon CM, Widerquist MAO, Lowery J. The updated Consolidated Framework for Implementation Research based on user feedback. Implementation Science. 2022;17(1):75.

59. Haynes A, Brennan S, Redman S, Williamson A, Gallego G, Butow P, et al. Figuring out fidelity: a worked example of the methods used to identify, critique and revise the essential elements of a contextualised intervention in health policy agencies. Implementation Science. 2016;11(1):23.

60. Ginsburg LR, Hoben M, Easterbrook A, Anderson RA, Estabrooks CA, Norton PG. Fidelity is not easy! Challenges and guidelines for assessing fidelity in complex interventions. Trials. 2021;22(1):372.

61. Hasson H, Blomberg S, Dunér A. Fidelity and moderating factors in complex interventions: a case study of a continuum of care program for frail elderly people in health and social care. Implementation Science. 2012;7(1):23.

62. Carroll C, Patterson M, Wood S, Booth A, Rick J, Balain S. A conceptual framework for implementation fidelity. Implementation Science. 2007;2(1):40.

63. Bellg, Borrelli B, Resnick B, Hecht J, Minicucci DS, Ory M, et al. Enhancing treatment fidelity in health behavior change studies: best practices and recommendations from the NIH Behavior Change Consortium. Health Psychol. 2004;23(5):443-51.

64. Weiner, Lewis CC, Stanick C, Powell BJ, Dorsey CN, Clary AS, et al. Psychometric assessment of three newly developed implementation outcome measures. Implement Science. 2017;12(1):108.

65. Hall A, Shoesmith A, Doherty E, McEvoy B, Mettert K, Lewis CC, et al. Evaluation of measures of sustainability and sustainability determinants for use in community, public health, and clinical settings: a systematic review. Implementation Science. 2022;17(1):81.

66. ImpleMentAll. Normalization Measure Development Questionnaire (short NoMAD). German Version: ImpleMentAll; 2018 [Available from: https://www.implementall.eu/NoMAD_German.pdf.

67. Naef R, Ernst J, Müeller M, Schmid-Mohler G. Translation and Psychometric Validation of the German Version of the Family Nursing Practice Scale (FNPS). Journal of Family Nursing. 2021;27(1):34-42.

For peer review only - http://bmjopen.bmj.com/site/about/guidelines.xhtml

68. Kien, Schultes, Szelag, Schoberberger, Gartlehner. German language questionnaires for assessing implementation constructs and outcomes of psychosocial and health-related interventions: a systematic review. Implementation Science. 2018;13(1):150.
69. https://www.project-redcap.org/. [Available from: https://www.project-redcap.org/.
70. Hamilton AB, Finley EP. Qualitative methods in implementation research: An introduction. Psychiatry Res. 2019;280:112516.
71. Ritchie J, Lewis J. Qualitative research practice. A Guide for Social Science Students and Researchers. London, Thousand Oaks, New Dehli: SAGE Publications; 2003. 331 p.
72. Finch T, Girling M, May C, Mair FS, Murray E, Treweek S, et al. Improving the normalization of complex interventions: part 2 - validation of the NoMAD instrument for assessing implementation work based on normalization process theory (NPT). BMC Medical Research Methodology. 2018;18(1):135.
73. Chambers DA, Glasgow RE, Stange KC. The dynamic sustainability framework: addressing the paradox of sustainment amid ongoing change. Implementation Science. 2013;8(1):117.
74. Neal JW, Neal ZP, VanDyke E, Kornbluh M. Expediting the Analysis of Qualitative Data in Evaluation: A Procedure for the Rapid Identification of Themes From Audio Recordings (RITA). American Journal of Evaluation. 2014;36(1):118-32.
75. Hsieh HF, Shannon SE. Three approaches to qualitative content analysis. Qualitative Health Research. 2005;15(9):1277-88.
76. Bengtsson M. How to plan and perform a qualitative study using content analysis. NursingPlus Open. 2016;2:8-14.
77. Bland M. An Introducation to Medical Statistics. Oxford: Oxford University Press; 2015.
78. Fetters MD, Molina-Azorin JF. The Journal of Mixed Methods Research Starts a New Decade: The Mixed Methods Research Integration Trilogy and Its Dimensions. Journal of Mixed Methods Research. 2017;11(3):291-307.
79. Palinkas LA, Aarons GA, Horwitz S, Chamberlain P, Hurlburt M, Landsverk J. Mixed method designs in implementation research. Administration and Policy in Mental Health 2011;38(1):44-53.
80. Zhang W, Creswell J. The use of "mixing" procedure of mixed methods in health services research. Medical Care. 2013;51(8):e51-7.
81. Mills T, Shannon R, O'Hara J, Lawton R, Sheard L. Development of a 'real-world' logic model through testing the feasibility of a complex healthcare intervention: the challenge of reconciling scalability and context-sensitivity. Evaluation. 2022;28(1):113-31.
82. World Medical Association. WMA declaration of Helsinki- Ethical Principles for medical research involving human subjects. World Medical Association; 2022.

For peer review only

For peer review only - http://bmjopen.bmj.com/site/about/guidelines.xhtml

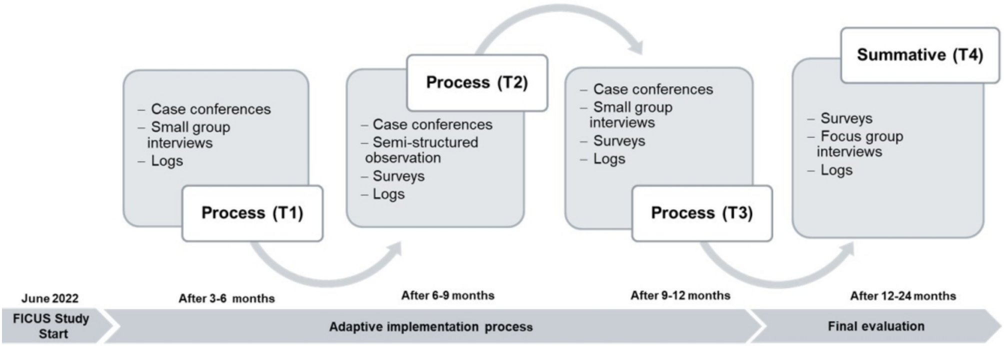

Implementation study overview

250x89mm (150 x 150 DPI)

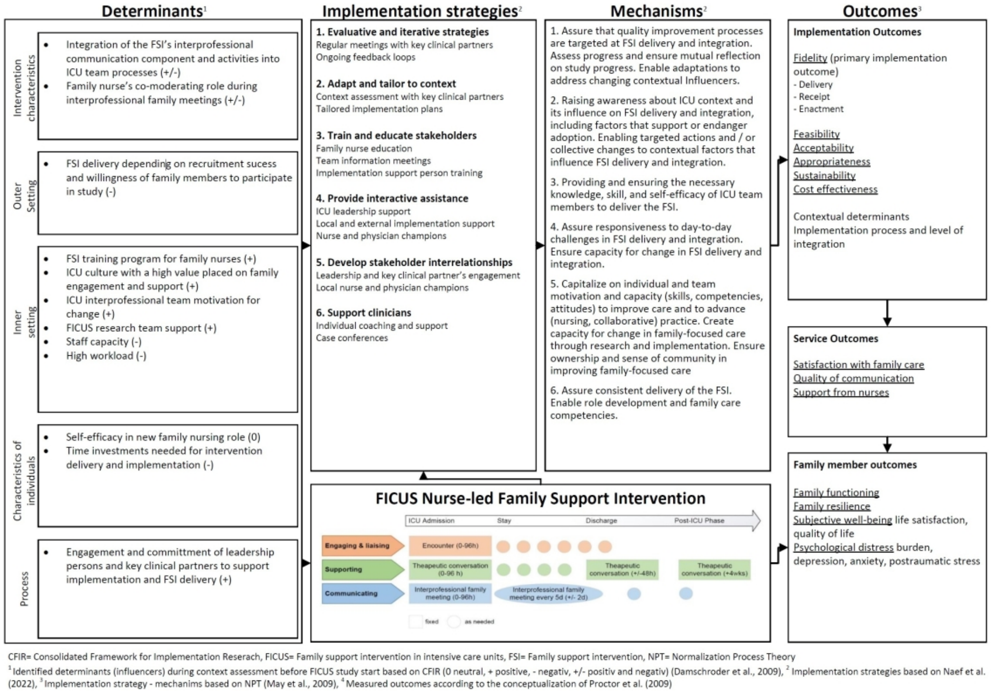

Implementation Research Logic Model

477x334mm (144 x 144 DPI)

Summary of the study endpoints, definition, data source operationalization and data collection timepoints

STUDY ENDPOINTS	DEFINITION	DATA SOURCE	OPERATIONALIZATION	DATA COLLECTION TIMEPOINT T1	T2	T3	T4
**Implementation Process**							
Process indicators	Course of integration of FSI in team / care delivery processes and observed integration mechanisms	KP	Small group interviews[1]	x		x	
		KP, HP	NoMAD [1, 2, 3]			x	x
		KP	Implementation rating tool [1]	x		x	
**Implementation Outcomes**							
**Fidelity: Delivery**							
Relative frequency	Number of interventions per length of stay (LoS)	-	Intervention fidelity log[1,9]	x	x	x	x
Absolute dose	Total minutes of interventions	-	Intervention fidelity log[1,9]	x	x	x	x
Consistency	Consistent delivery of the FSI within and across ICUs as defined per intervention manual	-	Intervention fidelity log[1,9]	x	x	x	x
Availability	Availability of FN (days) as defined per trial protocol to ensure intervention capacity	KP	Intervention capacity assessment[1]	x			
		FN	Focus group interviews[2]				x
**Fidelity: Receipt**							
Attendance at case conferences	Overall participation rate	FN, (LI)	Case conferences[1,4]	x	x	x	x
Comprehension & skills	Skills in working systematically with families	FN	FNPS[1, 5]	x	x		x
		FN	Focus group interviews[2]				x
**Fidelity Enactment**							
Engagement & performance	Quality and consistency of intervention performance	FN	Fidelity Self-Rating Tool[1]		x		x
		Researcher, FN	Semi-structured observation[1]		x		
		FN	Focus group interviews[2]				x
**Feasibility**	Extent to which the FSI can be successfully used and carried out within ICU settings	KP	FIM[1,2,6]	x		x	x
		KP, HP	Focus group interviews[2]				x
**Acceptability**	Perception among clinical partners that the FSI is agreeable	KP	AIM[1,2,7]	x		x	x
		KP, HP	Focus group interviews				x
**Appropriateness**	Perceived fit, relevance and compatibility of the FSI for clinical partners	KP, HP	IAM[1,2,8]	x		x	x
		KP, HP	Focus group interviews[2]				x
**Sustainability / Sustainment**	Existence of FN role assessed 6 and 12 months after study end How likely the FSI is to be sustained in the future with necessary structures and processes in place to maintain FSI in ICUs	KP	Sustainability implementation survey[10]				x
		KP; HP	Focus group interviews[2]				x
**Contextual Determinants**	Influencing barriers and facilitators as conceptualized in CFIR and NPT	KP	Small group interviews[1]	x		x	
		KP, HP	Focus group interviews[2]				x

For peer review only

Abbreviations: FN Family Nurses, LI local implementers, KP Key clinical partners, HP Health Care Professionals, ICU Intensive Care Unit, LoS Length of stay, CFIR Consolidated Framework for Implementation Research, NPT Normalization Process Theory

[1] Part of the process evaluation
[2] Part of the summative evaluation
[3] German Version of the Normalization Measure Development Questionnaire (NoMAD) (64)
[4] Monthly case conferences throughout the 18-months active implementation and delivery phase, attendance is mandatory for FN, voluntary for LI
[5] German version of the Family Nursing Practice Scale (FNPS) (65)
[6] German version of the Feasibility Intervention Measure (FIM) (66)
[7] German version of the Acceptability Intervention Measure (AIM) (66)
[8] German version of the Intervention Appropriateness Measure (66)
[9] Case report forms embedded in RED Cap (67) and completed throughout the study by family nurses / interventionists
[10] Data collection occurs 6 and 12 months after active implementation and intervention delivery / FICUS study end

For peer review only
